# Electrochemical Investigation of Compatibility Between Chloride‐ and Sulfide‐Based Solid Electrolytes Using Four‐Electrode Cells

**DOI:** 10.1002/smsc.70414

**Published:** 2026-10-08

**Authors:** Kotaro Yoshida, Atsunori Ikezawa, Peng Song, Shoma Yanagihara, Takuma Kasahara, Takeyoshi Okajima, Saneyuki Ohno, Hajime Arai

**Affiliations:** ^1^ School of Materials and Chemical Technology Institute of Science Tokyo Yokohama Kanagawa Japan; ^2^ Department of Applied Chemistry for Environment Tokyo Metropolitan University Hachioji Tokyo Japan; ^3^ Institute of Multidisciplinary Research for Advanced Materials Tohoku University Sendai Miyagi Japan; ^4^ Graduate School of Environmental Studies Tohoku University Sendai Miyagi Japan; ^5^ Department of Applied Chemistry Graduate School of Engineering Kyushu University Fukuoka Japan

**Keywords:** four‐electrode cell, heteroelectrolyte interface, hybrid bilayer cell, interface ionics, solid‐state ionics

## Abstract

The use of solid electrolytes (SEs) allows for the construction of solid‐state cells with bilayer‐to‐multilayer architectures. By appropriate choice of electrolytes for the positive‐ and negative‐electrode sides, electrolyte degradation can be mitigated, thus considerably increasing the design flexibility. However, certain electrolyte combinations lead to nonideal chemical compatibility, increasing the heteroelectrolyte interfacial resistance. Precise measurement of the interfacial resistance is necessary for quantitatively evaluating the compatibility of solid electrolytes. In this work, we employed a solid‐state four‐electrode cell to investigate the compatibility between three chloride‐based SEs—(Li_3_InCl_6_ (LIC), Li_3_ScCl_6_ (LSC), and Li_3_YCl_6_ (LYC))—and the sulfide‐based Li_7_P_2_S_8_I (LPSI). Electrochemical impedance spectroscopy (EIS) analysis reveals that the growth rate of the interfacial resistance is the highest in LIC, but the activation energies of the interfacial resistances are identical in all three SEs. Temperature‐dependent synchrotron X‐ray diffraction and density functional theory calculations strongly suggest that Li_4_PS_4_I, whose activation energy is similar to those measured by EIS, is the common interfacial product among the three cells, and that its relative degree of formation is consistent with the difference in interfacial resistance growth. This study demonstrates that electrochemical measurements using solid‐state four‐electrode cells constitute a promising technique for precisely investigating unstable heteroelectrolyte interfaces.

## Graphical abstract

Sulfide | chloride interfacial resistances and their evolution over time are measured using an all‐solid‐state four‐electrode cell. It is demonstrated that the interfacial resistances are governed by ion transport in the interphases formed by interfacial reactions.

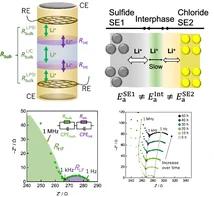

## 1 Introduction

The emergence of solid electrolytes (SEs) possessing superionic conductivity has enabled the development of high‐power solid‐state Li‐ion batteries (SSBs) [1, 2, 3, 4]. One of the most essential requirements of practical SEs is compatibility with active materials, as well as a broad electrochemical stability window (ESW) and high ductility [5]. A wide ESW is critical to ensure battery stability as significant reductive or oxidative decomposition of the SE at the active material | SE interface can trigger the formation of a resistive interphase [6, 7, 8, 9]. The ideal SE must also possess sufficient mechanical ductility to establish and sustain the electrolyte | active material interface, which functions as the reaction field, and the electrolyte | electrolyte interface, which provides the ion transport path, in order to accommodate the volume changes in the active material that can otherwise prevent effective contact among individual components. However, the lack of a ductile SE with a sufficiently wide ESW for both positive and negative electrodes [10, 11] makes it challenging to develop practical SSBs using a single SE.

A bilayer SE setup is a promising approach to prevent the degradation of SSBs due to SE decomposition [12, 13, 14, 15, 16, 17, 18, 19, 20, 21, 22]. Figure S1 presents a schematic illustration of the basic bilayer cell concept. In this configuration, a reduction‐resistant SE is placed in contact with the negative electrode material (Li metal), and an oxidation‐resistant SE is mixed with the positive electrode material [23]. The oxidation‐resistant SE also serves as the separator layer on the positive electrode side, thereby forming a heteroelectrolyte interface with another SE. Bilayer SE configurations have primarily been achieved with a sulfide‐ or oxide‐based SE on the negative electrode side and a halide‐based SE on the positive electrode side [16, 24]. In each case, however, the heteroelectrolyte interfaces strongly influence the increase in cell impedance. At thermodynamically stable oxide | sulfide interfaces, significant constriction resistance occurs because of the rigidity of the oxide‐based SE [13, 17]. In addition, residual carbonate and hydroxide impurities on the oxide‐based SE surface considerably increase the constriction resistance, making it challenging to develop bilayer cells using oxide‐based SEs [19]. The use of halide SEs, which possess both ductility and oxidation stability, is a promising strategy to address this issue. However, halide | sulfide interfaces are thermodynamically unstable, which leads to the formation of an interphase with poor ionic conductivity [24, 25]. A critical bottleneck in the development of a durable halide‐sulfide bilayer hybrid cell is the lack of a measurement method for precisely monitoring the evolution of interfacial resistance. Hence, it is essential to establish a method that investigates the growth of interfacial resistance between halide and sulfide SEs, thereby enabling the quantitative assessment of electrolyte compatibility and the evaluation of interfacial physicochemical factors such as activation energy [26].

Electrochemical impedance spectroscopy (EIS) using two‐electrode cells has been primarily reported for evaluating interfacial resistance at the interface, for example, halide | sulfide interface. However, the halide | sulfide interfacial resistance overlaps with the electrode | electrolyte processes having similar time constants, inhibiting clear isolation of individual processes. Our group recently reported a solid‐state four‐electrode cell for precisely investigating the interfacial resistance between different sulfide‐based SEs by EIS [27]. In this configuration, two mesh‐shaped Li_7_Ti_5_O_12_ | Li_4_Ti_5_O_12_ (R‐LTO) reference electrodes [28] are positioned near the SE | SE interface, allowing us to focus on the interfacial resistance between the two sulfide‐based SEs. This method is considered suitable for isolating the interfacial resistance at heteroelectrolyte interfaces, for example, halide | sulfide interfaces, and for analyzing the interfacial resistance growth and activation energy of interfacial ion transport.

In this study, we investigated the electrochemical/chemical compatibility between halide and sulfide SEs by analyzing the interfacial resistance using electrochemical four‐electrode cells. Li_7_P_2_S_8_I with a composition of Li_2_S‐P_2_S_5_‐LiI (LPSI) was selected as the sulfide SE because of its high ionic conductivity and sufficient stability against In–Li alloys [29, 30, 31]. Li_3_InCl_6_ (LIC) [32], Li_3_ScCl_6_ (LSC) [33], and Li_3_YCl_6_ (LYC) [34] were selected as halide SEs because they have high ionic conductivity, deformability, and similar compositions, which may be conducive to identifying the origin of the reactivity. Temperature‐dependent synchrotron X‐ray diffraction (SXRD) measurements were conducted to detect the interfacial compounds generated by the reaction between the SEs. The reaction energies for the possible reactions were calculated using density functional theory (DFT), and the results were compared with those obtained from electrochemical measurements. The interfacial resistance growth was more significant in LIC than in LSC and LYC; this trend corresponded to the reactivity of this chloride SE toward LPSI, as suggested by the SXRD data and the thermodynamic stability estimated by DFT calculations. Furthermore, the activation energy for ion transport within the interphase matched that of Li_4_PS_4_I, which is suggested by SXRD as one of the interfacial reaction products. These results strongly suggested that ion transport at the chloride | sulfide interface is restricted by the low‐conductivity interphase formed by interfacial reactions and that Li_4_PS_4_I, which has higher ionic conductivity, forms an ion transport pathway within the interphase. This study demonstrates that the electrochemical four‐electrode cell is suitable for the direct and sensitive evaluation of interfacial compatibility and for elucidating the mechanism underlying interfacial ion transport.

## 2 Methods

### 2.1 Material Preparation and Characterization

The glass‐ceramic Li_7_P_2_S_8_I (LPSI) was synthesized by mechanochemical milling of Li_2_S: P_2_S_5_: LiI = 60: 20: 20 mol% (rotating speed: 370 rpm, rotating period: 60 min, interval: 10 min, number of total cycles: 38) using a planetary ball mill (Pulverrisette 7 Classic Line, FRITSCH), followed by heating at 473 K for 3 h in a vacuumed silica tube using an electric furnace (KDF‐S70, Denken‐Highdental) [31]. LIC, LSC, and LYC were synthesized by mechanochemical milling of LiCl: InCl_3_, ScCl_3_, and YCl_3_ 75:25 mol% (rotating speed: 500 rpm, rotating period: 15 min, interval: 5 min, and number of total cycles: 99) [21]. The R‐LTO powders, Li_4_Ti_5_O_12_: Li_7_Ti_5_O_12_ = 33:67 mol%, and Li–In alloy films, Li: In = 25:75 at%, were prepared by the chemical lithiation of Li_4_Ti_5_O_12_ powder (Ishihara Sangyo) and an In film (9 mm in diameter and 50 μm in thickness, Nilaco) following our previously reported method [27, 28].

X‐ray diffraction (XRD) and ionic conductivity measurements were performed to characterize the SEs. XRD measurements were performed using an X‐ray diffractometer (SmartLab, Rigaku) with Cu Kα1 radiation. The SEs were sealed in an Ar‐filled container during the measurements. The scan rate, step, and range were 4° min^–1^, 0.01°, and 2*θ* = 0°–60°, respectively. The ionic conductivities of the SEs were evaluated by AC impedance measurements using electrochemical two‐electrode cells comprising Ti powder electrodes and SEs (Figure S2). First, the SE powder was pressurized in a stainless steel mold at 140 MPa for 1 min to obtain SE pellets with a diameter of 10 mm and a thickness of 1.0 mm. Then, Ti powder was cast on each side of the pellet and pressurized at 280 MPa using a torque wrench in an Ar‐filled airtight cell. Finally, AC impedance measurements were performed using a multichannel electrochemical measurement system (VSP‐300, BioLogic) and a constant‐temperature oven (SH‐222, Espec) set at 263–298 K. The AC amplitude and frequency range were 10 mV and 7 MHz–50 mHz, respectively.

The XRD patterns of LPSI, LIC, LSC, and LYC are shown in Figure S3a–d. LPSI shows a broad diffraction pattern, with prominent peaks at around 20° and 30°, which were attributed to the highly conductive phases. In addition, the peaks around 21° and 27.9° were assigned to Li_4_PS_4_I, which possesses lower ionic conductivity [30, 31, 35]. LIC, LSC, and LYC exhibit distinct XRD patterns corresponding to each SE. Table S1 shows the measured and reported [21, 30, 31, 32, 34, 36] ionic conductivities at 298 K and the activation energies of each SE. The measured values agreed well with the reported data.

### 2.2 Electrochemical Measurements

Figure S4 shows a schematic of an electrochemical four‐electrode cell used to measure the interfacial resistances between the chloride SEs and LPSI. The cell was composed of R‐LTO reference electrodes, Li–In counter electrodes, chloride SE, and LPSI powder. The R‐LTO reference electrodes were fabricated using the method reported in our previous work [27]. The R‐LTO composite (R‐LTO: poly(vinylidene difluoride) = 86.5:13.5 wt%) was cast onto the center (*φ* = 5 mm) of a Ni mesh (3Ni7‐3/0, Taiyo Wire Cloth, aperture ratio: 90%–91%, thickness: 0.08 mm, *φ* = 10 mm) current collector. The four‐electrode cells were fabricated as follows. First, 100 mg of LPSI powder was placed in a poly(ethylene terephthalate) cylinder. The R‐LTO reference electrode was placed on the LPSI powder, and then 100 mg of LPSI powder was again placed on the R‐LTO reference electrode to obtain a sandwich structure. Next, 50 mg of the chloride SE was placed on the LPSI powder to form an interface. Another interface was formed by placing 100 mg of LPSI powder on the chloride SE, thereby sandwiching it with the LPSI powder. Then, the R‐LTO reference electrode was again placed on the LPSI powder, and 100 mg of LPSI powder was placed on top of the reference electrode to sandwich it. The powder‐filled cylinder was pressurized with 280 MPa for 1 min using a uniaxial hydraulic press. Li–In foils were placed on the outer sides of the stacked pellet to serve as counter electrodes. Finally, the cell was pressurized to approximately 20 MPa using a torque wrench and sealed in a plastic container filled with Ar gas. The thicknesses of the LPSI layer incorporating R‐LTO and the chloride SEs were ca. 1.6 and ca. 0.3 mm, respectively. The cell was fabricated at 298 K in an Ar‐filled glove box.

AC impedance measurements were conducted at the open‐circuit voltage with a frequency range of 7 MHz–50 mHz and an AC amplitude of 10 mV by using a multichannel electrochemical measurement system and a constant‐temperature oven. Curve fitting of the impedance spectra was performed using commercial software (Z‐View, Scribner). The capacitances (*C*) were calculated using the following equation [37]:

(1) $$C=\;\frac{{\left(Q\times R\right)}^{\frac{1}{\alpha }}}{R}$$

where *Q* is the constant phase element (CPE) constant, *α* is the CPE index, and *R* is the resistance.

### 2.3 Characterization of Reaction Products at Chloride SE | LPSI Interfaces

Temperature‐dependent SXRD measurements were performed at SPring‐8 BL02B2 to identify the reaction products at the chloride | LPSI interfaces. Equal amounts of LPSI and the chloride SE powder were mixed in an agate mortar for 15 min, and the mixture was loaded into a vacuum‐sealed quartz capillary with an inner diameter of 0.3 mm. First, SXRD analysis was performed at 300 K for 3 min. Then, the capillary was heated up to 473 K at a heating rate of 20 K min^–1^. Next, SXRD analysis was performed at 473 K for 3 min, followed by measurements at 300 K for 30 s. The wavelength used for the measurements was determined to be 0.413965(4) Å by calibration against CeO_2_ as a standard sample. The calibration was performed using Rietveld analysis software (Z‐Rietveld) [38, 39].

### 2.4 DFT Calculations

The thermodynamic stability of Li_3_XCl_6_ (X = In, Sc, or Y) was determined by calculating its energy above the convex hull (*E*_hull_) using the Pymatgen library [40]. Thermodynamically relevant phases containing the constituent elements, except Li_3_YCl_6,_ were obtained from the materials project (MP) database [41], and the corresponding phase diagrams were constructed. Since Li_3_YCl_6_ was unavailable in the MP database, we performed first‐principles calculations to determine its total energy. Specifically, DFT calculations were conducted using the Vienna ab initio simulation package (VASP) [42, 43] with projector‐augmented wave potentials [44] and the Perdew–Burke–Ernzerhof (PBE) exchange‐correlation functional [45]. Structural optimization was performed using a plane‐wave cutoff energy of 600 eV and an 8 × 8 × 6 k‐point mesh. To maintain consistency with the MP database, an anion correction was applied to account for the energy contribution of Cl^‒^ ions [46].

The electrochemical stability of Li_3_XCl_6_ was assessed by evaluating its ESW relative to Li/Li^+^ [47]. The potential redox reactions occurring at the SE–Li interface were considered in this evaluation. The redox reaction energy (Δ*E*_redox_) was calculated using the following equation:

$$\begin{array}{cc} \Delta E_{\text{redox}}(\text{phase},\;x) & =\;E_{\mathrm{tot}}[(1-x)c_{\text{phase}}\;+\;xc_{\mathrm{Li}}]\;\\ & \;-\;[(1-x)E_{\mathrm{tot}}(c_{\text{phase}})\;+\;xE_{\mathrm{tot}}(c_{\mathrm{Li}})] \end{array}$$

where *c*_Li_ and *c*_phase_ denote the compositions of lithium metal and SEs, respectively, and *x* represents the molar fraction of lithium. The corresponding equilibrium potential (*ϕ*_eq_) was subsequently determined as:

(2) $$\phi_{\mathrm{eq}}\;=\;-\frac{\Delta E_{\text{redox}}}{x}$$

The chemical stability at the interface between Li_3_XCl_6_ and Li_7_P_2_S_8_I was evaluated on the basis of the interfacial reaction energy with potential decomposition products, as developed by Ceder et al. [48]. Because of the absence of the Li_7_P_2_S_8_I and Li_4_PS_4_I phases in the Materials Project database and given that experimentally observed phases are typically amorphous [49], their energies were approximated by the summed energies of the constituent Li_2_S, P_2_S_5_, and LiI phases. If the deviations of the actual amorphous energies from the crystalline references are denoted by δ_7_ and δ_4_ for Li_7_P_2_S_8_I and Li_4_PS_4_I, respectively, the calculated reaction energies would include an unknown combination of these offsets. The magnitude of these deviations cannot be determined within the present model; therefore, the absolute reaction energies and small differences between the product sets are interpreted qualitatively. The interfacial chemical reaction energy ($\text{Δ}E_{\mathrm{chem}}$) was calculated to identify the reaction products with the most favorable thermodynamic conditions at various formula‐unit‐based molar fractions (*x*):

(3) $$\begin{array}{cc} \text{Δ}E_{\mathrm{chem}}(x) & =E_{\mathrm{tot}}(\text{interface products},x)\\ & \;-[(1-x)E_{\mathrm{tot}}({\mathrm{Li}}_{3}{\mathrm{XCl}}_{6})+xE_{\mathrm{tot}}({\mathrm{Li}}_{7}P_{2}S_{8}I)] \end{array}$$

where *E*_tot_ (interface products, *x*) represents the total energy of equilibrium phases formed at the interface.

## 3 Results and Discussion

### 3.1 Measurement of Ion Transport Resistance at Chloride | LPSI Interfaces

Using the solid‐state four‐electrode cell, we successfully measured the chloride | LPSI interfacial resistances and their activation energy. The results showed that ion transport in the interphase formed during the interfacial reaction is the rate‐limiting process at the chloride | sulfide interface.

Electrochemical measurements performed using a four‐electrode configuration enable the exclusive detection of ion transport processes occurring between the reference electrodes while ignoring those at the counter electrodes. Within the region between the reference electrodes, the primary ion transport processes include the bulk transport process (*R*_bulk_) in each SE and the interfacial process between the SEs (*R*_int_). In the LIC | LPSI cell, the transport processes include *R*_bulk_, which involves all the SEs, and *R*_int_, as shown in Figure 1a. Thus, to isolate the interfacial transport process from the bulk, EIS was performed using a four‐electrode cell. Figure 1b shows the impedance spectrum of the four‐electrode cell with the LIC layer sandwiched between the LPSI layers, measured after 1 h of cell assembly.

**FIGURE 1:**
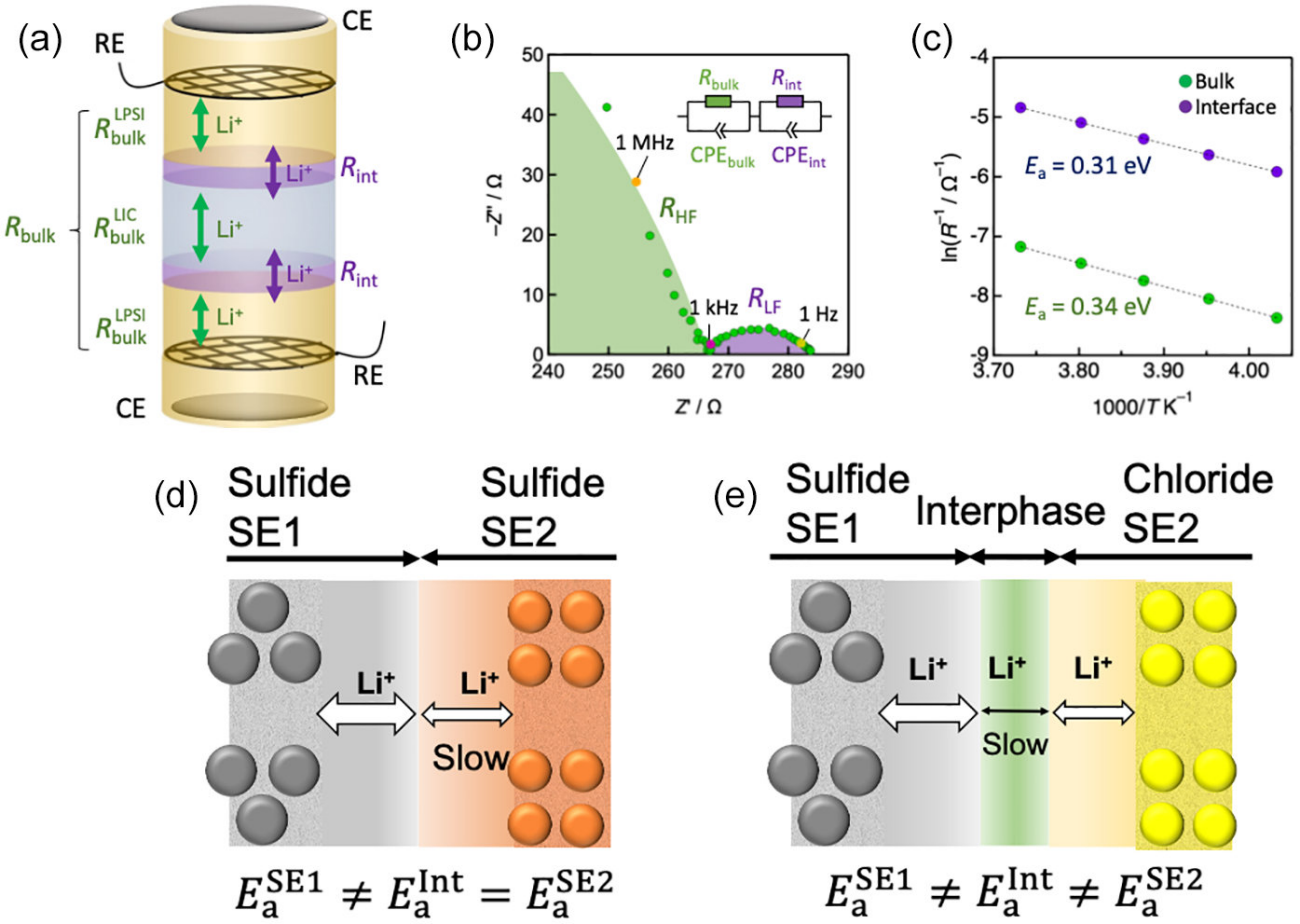
(a) A schematic of the solid‐state four‐electrode cell with a heteroelectrolyte interface. (b) Impedance spectra of the Li_3_InCl_6_ | Li_7_P_2_S_8_I cell. (c) Arrhenius plots of *R*_bulk_ and *R*_int_ measured with the Li_3_InCl_6_ | Li_7_P_2_S_8_I cell. Proposed difference in the rate‐determining process between the (d) sulfide | sulfide interface and (e) sulfide | chloride interface.

The impedance spectra obtained using the four‐electrode cells with LSC and LYC are shown in Figure S5a,b. Hereafter, we refer to these cells as LIC | LPSI, LSC | LPSI, and LYC | LPSI cells. The impedance spectra of the cells exhibit two distinct semicircles, one above 1 kHz (*R*_HF_) and the other around 10 Hz (*R*_LF_). We previously used four‐electrode cells to investigate sulfide‐based heteroelectrolyte interfaces and observed impedance spectra with two comparable semicircles with time constants on the order of 10^–8^–10^–9^ s and 10^–2^ s, assigned to *R*_bulk_ and *R*_int_, respectively [27]. The parameters obtained from curve fitting using the equivalent circuit shown in the inset of Figure 1b are presented in Table 1. *R*_HF_ and *R*_LF_ observed in this study can also be attributed to *R*_bulk_ and *R*_int_, given the similarity of the time constants. Hereafter, we refer to *R*_HF_ as *R*_bulk_ and *R*_LF_ as *R*_int_. As shown in Figure 1a, since two interfaces are formed between the reference electrodes, *R*_int_ is the sum of their resistances.

**TABLE 1:** Fitting parameters obtained by the curve fittings.

Cell	Component	*R*, Ω	CPE constant, F s^*p *– 1^	CPE index, *p*	Capacitance, F	Time constant, s
LIC|LPSI	P1	247.4	1.2 × 10^–10^	0.98	8.4 × 10^–11^	2.1 × 10^–8^
	P2	15.7	2.0 × 10^–3^	0.67	3.6 × 10^–4^	5.7 × 10^–3^
LSC|LPSI	P1	209.9	4.3 × 10^–9^	0.77	6.7 × 10^–11^	1.4 × 10^–8^
	P2	20	3.2 × 10^–3^	0.60	5.1 × 10^–4^	1.0 × 10^–2^
LYC|LPSI	P1	465.6	3.4 × 10^–10^	0.92	8.7 × 10^–11^	4.1 × 10^–8^
	P2	23.6	1.1 × 10^–2^	0.48	2.6 × 10^–3^	6.0 × 10^–2^

To evaluate the activation energy (*E*_a_) for each process, the temperature dependence of the corresponding resistance was measured over the range of 273–293 K (Figures S6–S8). Figure 1c shows the Arrhenius plots for *R*_int_ and *R*_bulk_ obtained with the LIC | LPSI cell. The Arrhenius plots for the LSC | LPSI and LYC | LPSI cells are shown in Figure S9a,b, respectively. *R*_int_ and *R*_bulk_ show Arrhenius‐type temperature dependencies; thus, the *E*_a_ value of each process can be calculated from the slopes using the Arrhenius equation as shown in the inset of the graphs. The *E*_a_ value of *R*_int_ is almost identical (0.30–0.31 eV) among all cells. Notably, the *E*_a_ value for *R*_int_ in each cell does not correspond to the *E*_a_ of any chloride SE (*E*_a_ = 0.35–0.40 eV) or LPSI (*E*_a_ = 0.34 eV). We have demonstrated that at sulfide‐based heteroelectrolyte interfaces, *E*_a_ of interfacial ion transport corresponds to that of the SE with lower conductivity (Figure 1d) [27]. This is because Li^+^ transport to or from the low‐conductivity SE is the rate‐limiting process in interfacial Li^+^ transport. On the other hand, the measured *E*_a_ values of the interfacial process are significantly lower than those of the employed chloride and sulfide electrolytes. This indicates the formation of an interphase that conducts ions with a lower *E*_a_ and with inferior conductivity as compared to those of the employed chloride and sulfide SEs (Figure 1e). This result provides additional information about the interface and interphases that are accessible from the four‐electrode measurements.

Since the CPE index of P_2_ is low (0.48–0.67), the interfacial resistances measured in this study may include not only the resistance of the interphase but also other resistances such as those derived from the space charge layer and current constriction. On the other hand, *E*_a_ differs from that of the SEs forming the interface, and the interfacial resistance clearly increases over time, as described in Section 3.2. Hence, we concluded that the interphase resistance predominantly constitutes interfacial resistance.

### 3.2 Comparison of Growth Rates of Interfacial Resistance

Using the four‐electrode measurement setup, the continuous evolution of the chloride | sulfide interfacial resistance is quantitatively examined at room temperature (298 K). It was found that the growth of the interfacial resistance increased in the order LIC > LSC > LYC.

The time variation of the impedance spectra of the LIC | LPSI cell at room temperature (298 K) is shown in Figure 2a. The impedance spectra of the LSC | LPSI, LYC | LPSI, and LPSI | LPSI cells under the same conditions are shown in Figure S10. In the chloride | LPSI cells, particularly the LIC | LPSI cell, increases in *R*_bulk_ and *R*_int_ can be observed. To compare the magnitude of these increases, the increase rates of the resistances at 50 h (*R*_*t*__= 50 h_/*R*_*t*__= 0 h_) were compared. The LPSI | LPSI cell shows the lowest *R*_int_ growth rate of 1.1. Since the interface between identical SEs is stable, this increase is attributed to changes in the interface contact state due to factors such as pressure relaxation. The *R*_int_ growth rate of the LIC | LPSI cell (2.2) is twice that of the LPSI | LPSI cell, indicating the continuous growth of a high‐resistivity interphase due to interfacial reactions and lower compatibility at the LIC | LPSI interface. The *R*_int_ increase rate of the LSC | LPSI cell is approximately 1.3 times higher (1.4) than that of the LPSI | LPSI cell but almost 40% lower than that of the LIC | LPSI cell. This trend indicated that the LSC | LPSI interface shows better compatibility than the LIC | LPSI interface. Furthermore, the *R*_int_ increase rate of the LYC | LPSI cell (1.3) is approximately 7% lower than that of the LSC | LPSI cell, indicating slightly greater compatibility of the LYC | LPSI interface. However, this subtle difference might include experimental uncertainty as each cell was prepared only once.

**FIGURE 2:**
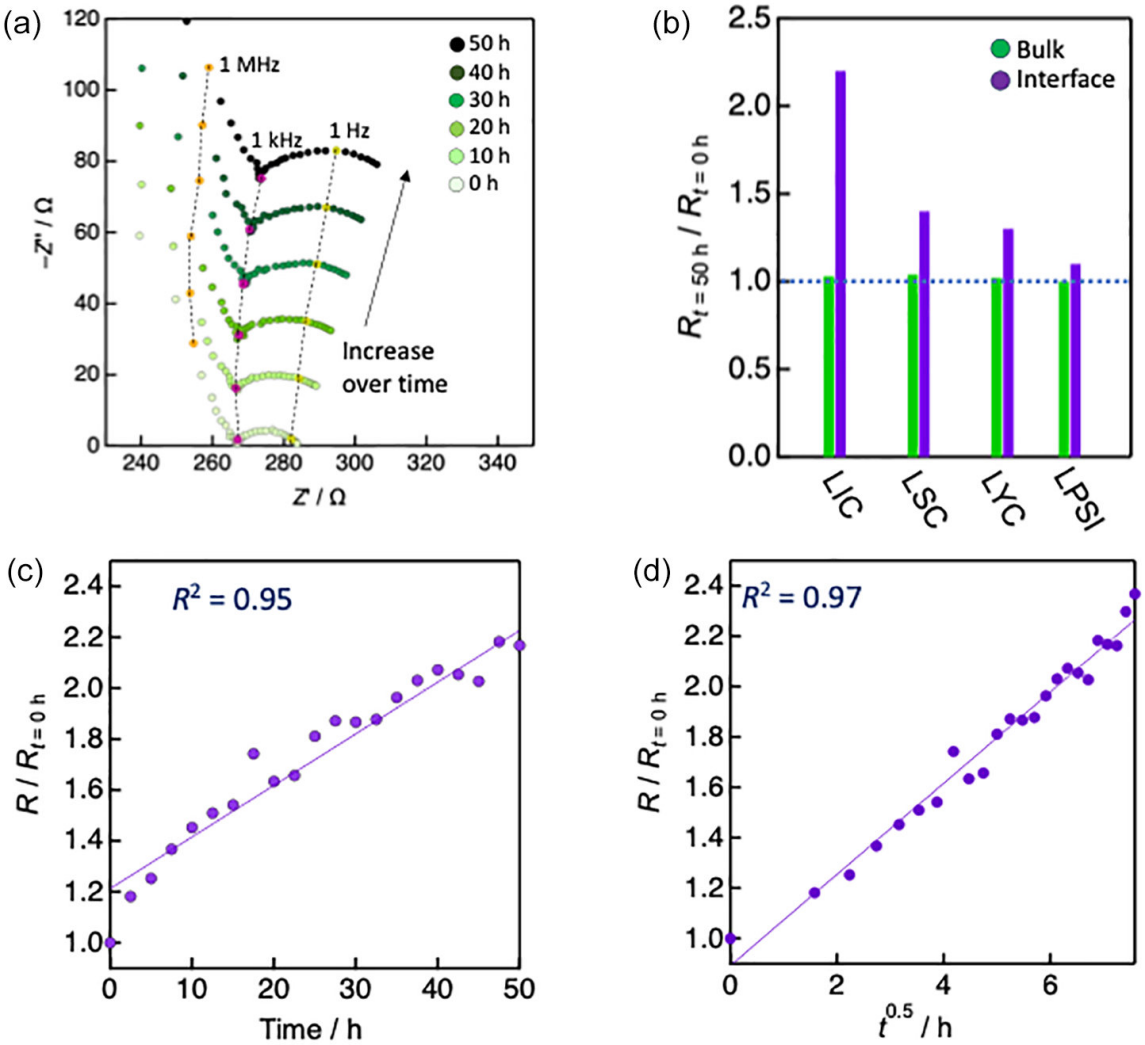
(a) Time variation of the impedance spectrum of the Li_3_InCl_6_ | Li_7_P_2_S_8_I cell at room temperature (298 K). (b) Resistance growth rates at 50 h (*R*_*t*__= 50 h_/*R*_*t*__= 0 h_). Interfacial resistance growth rate of the Li_3_InCl_6_ | Li_7_P_2_S_8_I cell plotted against (c) time and (d) square root of time.

Although an increase in *R*_bulk_ is also observed in the chloride | LPSI cells, the rate of increase is below 1.04, that is, one order of magnitude smaller than that of *R*_int_. In addition, the magnitude of the *R*_bulk_ increase rates (LSC (1.04) > LIC (1.03) > LYC (1.02)) followed a different order from that of *R*_int_. These results suggest that the observed increase in *R*_bulk_ stemmed from factors unrelated to interfacial reactions, such as pressure relaxation.

When the interfacial reaction is diffusion‐limited, the increase in interfacial resistance is proportional to the square root of time [50]. Figure 2c,d shows the *R*_int_ of the LIC | LPSI cell plotted against time and the square root of time. The corresponding graphs for the LSC | LPSI and LYC | LPSI cells are shown in Figure S11. The approximate line and its coefficient of determination (*R* ^2^) are shown in the graphs. The plot of *R*_int_ of the LIC | LPSI interface against time shows less linearity (*R* ^2^ = 0.95) as compared to the plot of *R*_int_ against the square root of time (*R* ^2^ = 0.97), suggesting that the interfacial reaction is diffusion‐controlled. On the other hand, the *R*_int_ values of the LSC | LPSI and LYC | LPSI interfaces exhibited greater linearity when plotted against time (*R* ^2^ = 0.98 and 0.92) than when plotted against the square root of time (*R* ^2^ = 0.92 and 0.89, respectively). This result suggests that the interfacial reaction is rate‐limited by processes other than diffusion, such as migration or chemical reactions. There are three possible causes underlying the deviation in the rate‐limiting factor: (1) differences in the interdiffusion coefficients of ions, (2) differences in the morphology of the reaction front, and (3) differences in the reaction kinetics at the reaction front. Although it is currently impossible to identify the primary cause among these, the results of DFT analysis (Section 3.3) partially support the third reason. DFT analysis reveals that interfacial reactions become thermodynamically favorable in the order LIC > LSC > LYC. If these reactions are similar, the linear free‐energy relationship predicts that the reaction rates at the reaction front increase in the order LIC > LSC > LYC. Therefore, it is possible that the reaction was diffusion‐limited for LIC and reaction‐limited for LSC and LYC. For a more detailed discussion, it is necessary to analyze the reaction kinetics and interfacial morphology.

### 3.3 Analysis of Interphase Species by Temperature‐Dependent SXRD

The unheated mixture showed no temporal change in the XRD pattern (Figure S12). Therefore, temperature‐dependent synchrotron XRD analysis was performed to investigate the interfacial reaction products, assuming that heat treatment would facilitate the interfacial reaction. Figure 3a–c shows the SXRD patterns measured at 300 K before and after the temperature increase to 473 K. Reference SXRD patterns simulated using Z‐Rietveld [38, 39] are also included. For all the samples, diffraction patterns attributed to Li_4_PS_4_I, P_4_S_3_, LiCl, and LiI are observed after the temperature increase, suggesting that these compounds are included in the interfacial reaction products. In addition, peaks due to Li_4_PS_4_I and LiCl were detected in the laboratory XRD pattern after calcination at 423 K for 72 h (Figure S13), indicating that these compounds are likely to be interfacial reaction products. Notably, the Li^+^ conductivity of Li_4_PS_4_I (0.03–0.47 mS cm^–1^) [51, 52, 53], one of the major products, was higher than that of the other reaction products but about one order of magnitude lower than those of LPSI, LIC, LSC, and LYC. Moreover, Li_4_PS_4_I with nanometer‐sized crystallites has been reported to show *E*_a_ values of 0.29–0.35 eV, which are comparable to those of *R*_int_ (~0.31 eV) [53]. These results strongly suggest that Li_4_PS_4_I creates an ion‐conducting pathway in the low‐conductivity chloride | LPSI interphase formed by the interfacial reaction.

**FIGURE 3:**
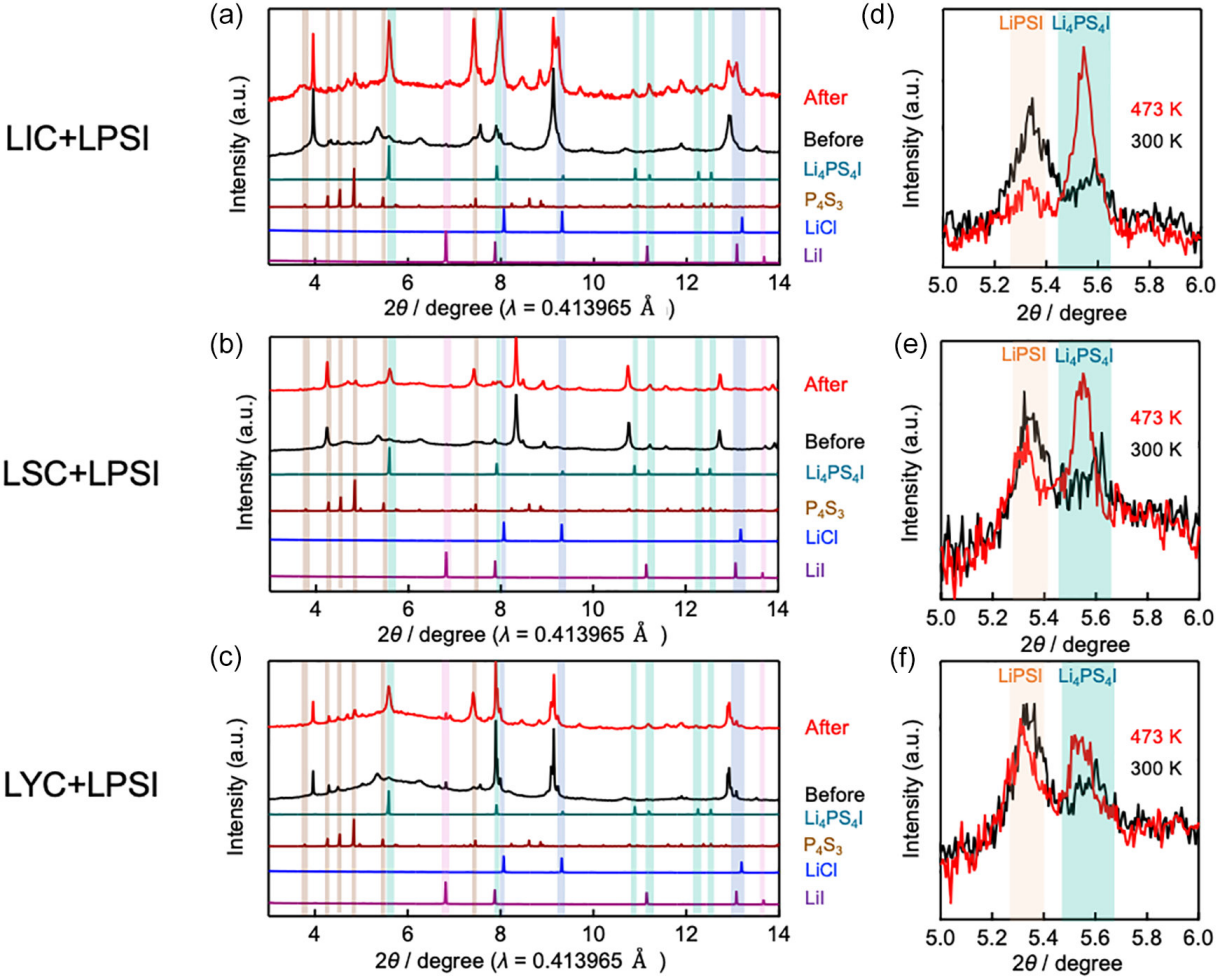
SXRD patterns of (a) Li_3_InCl_6_ + Li_7_P_2_S_8_I, (b) Li_3_ScCl_6_ + Li_7_P_2_S_8_I, and (b) Li_3_YCl_6_ + Li_7_P_2_S_8_I before and after the temperature increase to 473 K. SXRD patterns of (d) Li_3_InCl_6_ + Li_7_P_2_S_8_I, (e) Li_3_ScCl_6_ + Li_7_P_2_S_8_I, and (f) Li_3_YCl_6_ + Li_7_P_2_S_8_I at 300 and 473 K.

While XRD measurements analyzed the reaction products at temperatures above 423 K, electrochemical measurements analyzed the reaction products at room temperature. Since the reaction rates and crystal growth rates are lower at room temperature, amorphous phases, nanocrystals, and metastable phases that are not observed in the XRD measurements may be formed. In fact, the *E*_*a*_ value for interfacial resistance matches that of nanocrystalline LPSI rather than that of the bulk material.

Subsequently, the reactivity of each chloride SE toward LPSI was qualitatively compared by analyzing the diffraction peaks. Figure 3d–f shows the SXRD patterns at 300 and 473 K during the temperature increase test. The diffraction peak at 5.3°, which decays with increasing temperature, is attributed to LPSI, while the diffraction peak at 5.5°, which increases with increasing temperature, is ascribed to Li_4_PS_4_I. The integrated intensity ratio of the diffraction peaks of LPSI relative to Li_4_PS_4_I at 473 K follows the order LIC + LPSI (0) < LSC + LPSI (0.214) < LYC + LPSI (0.812) (Table 2), indicating that the reactivity toward LPSI is higher in the order of LIC > LSC > LYC. This trend agrees with the *R*_int_ growth rate derived from the electrochemical measurements, suggesting that differences in the interfacial reaction kinetics among the chloride SEs account for the difference in the *R*_int_ growth rate.

**TABLE 2:** Integrated intensity of the diffraction peaks of LPSI (*I*_LPSI_) and Li_4_PS_4_I ($I_{{\mathrm{Li}}_{4}{\mathrm{PS}}_{4}I}$) and integrated intensity ratio ($I_{\mathrm{LPSI}}/I_{{\mathrm{Li}}_{4}{\mathrm{PS}}_{4}I}$) at 473 K.

	LPSI + LIC	LPSI + LSC	LPSI + LYC
$I{\mathrm{Li}}_{4}{\mathrm{PS}}_{4}I/\mathrm{cps}{}^{\circ}$	1380	154	444
$I_{\mathrm{LPSI}}/\mathrm{cps}{}^{\circ}$	—	721	547
$I_{\mathrm{LPSI}}/I_{{\mathrm{Li}}_{4}{\mathrm{PS}}_{4}I}$	0	0.214	0.812

Li_4_PS_4_I is known to be formed as a by‐product of LPSI upon heating at high temperatures [30]. To confirm whether Li_4_PS_4_I observed here is the reaction product, the LPSI powder was heated at 423 K for 72 h. Figure S3a shows the XRD patterns of LPSI before and after heating. Although small peaks due to Li_4_PS_4_I were identified prior to heating, their intensities showed minimal change after heating. These results led us to conclude that the interfacial reaction between the chloride SE and LPSI generated the majority of Li_4_PS_4_I.

### 3.4 Calculation of Interfacial Reaction Energies by DFT

DFT calculations were performed to thermodynamically verify the difference in reactivity between the chloride SEs and LPSI. Ternary convex hull analysis reveals that LIC is thermodynamically stable, whereas LSC and LYC are metastable, located 14 and 24 meV/atom above the hull, respectively (Figure 4a–c). Specifically, the Li–In–Cl system featured many stable Li–In alloys, enabling Li_3_InCl_6_ to decompose into InCl_2_ and LiCl at 2.64 V during electrochemical reduction, followed by the formation of Li–In alloys upon further reduction (Figure S14). In contrast, the Li–Sc–Cl and Li–Y–Cl systems lack the thermodynamically stable Li–Sc and Li–Y alloys; hence, LSC and LYC decompose only into their chlorides upon reduction, as shown in the following reactions: 5Li_3_ScCl_6_ + 7Li → 22LiCl + Sc_5_Cl_8_ (0.91 V) and 2Li_3_YCl_6_ + 3Li → 9LiCl + Y_2_Cl_3_ (0.65 V). Upon oxidation, all three compounds initially generate XCl_3_ and Cl_2_, leading to similar oxidation voltages of about 4.3 V. Notably, LPSI oxidizes at 2.55 V to form LiI, P_2_S_5_, and S. Thus, the mismatch in the ESWs between LPSI and LIC may induce interfacial decomposition during battery cycling.

**FIGURE 4:**
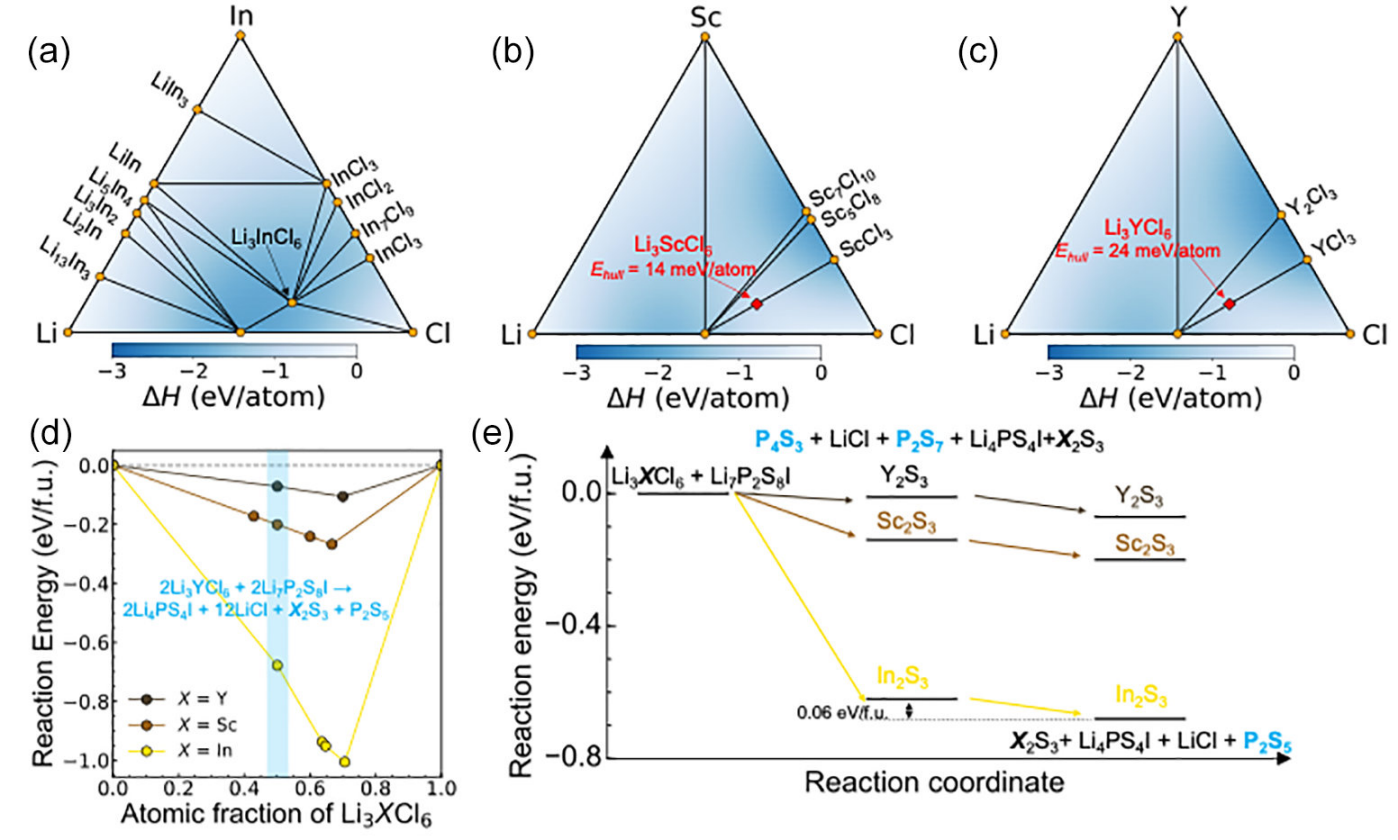
Phase diagrams of (a) LIC, (b) LSC, and (c) LYC, where thermodynamically stable phases are marked with orange circles and unstable phases with red diamonds. (d) Phase equilibria and minimum mutual decomposition energies at the interfaces between LXC and LPSI. (e) Reaction energies considering the formation of intermediate phases.

We further investigated the interfacial stability of LXC with LPSI, as shown in Figure 4d. The thermodynamic equilibria are summarized in Table S2. Lower values indicate more severe interfacial reactions and thus poorer stability. The absolute values of interfacial reaction energy (|∆*E*_chem_|) follow the order LIC > LSC > LYC across all calculated formula‐unit‐based molar fractions, indicating that the thermodynamic interfacial stability is the lowest for LIC | LPSI and the highest for LYC | LPSI. The reaction energy reaches a minimum when the formula‐unit‐based molar fraction of LXC is between 0.6 and 0.8; however, products such as LiInS_2_, which are predicted to be formed within this range and were detected in solid‐state Li–S battery cathodes in a previous study [21], are not detected experimentally in the present study. This discrepancy indicates that static DFT calculations cannot quantitatively predict the experimentally accessible product assemblage. The calculations describe crystalline‐phase equilibria based on static energies at 0 K, whereas the electrochemical measurements were performed near room temperature, and the SXRD products were observed after heating the initially disordered mixed powders to 473 K. Therefore, finite‐temperature contributions, interfacial effects, structural disorder, kinetic limitations, local compositional heterogeneity, and experimental detection limits may influence the observed products. For the model composition at a formula unit‐based molar fraction of 0.5, the calculated product set contains Li_4_PS_4_I, LiCl, and P_2_S_5_. In contrast, SXRD analysis revealed the presence of Li_4_PS_4_I, LiCl, and P_4_S_3_, whereas P_2_S_5_ was not detected. The 0 K P–S phase diagram in Figure S15 provides qualitative information about the phase stability. For the LIC system, further conversion of the P_4_S_3_‐containing product set to the P_2_S_5_‐containing product set changes the static reaction energy by only −0.06 eV/f.u. This small thermodynamic difference is not a kinetic barrier and cannot by itself explain the experimental observation or persistence of P_4_S_3_. Accordingly, the calculations are used only to compare the qualitative thermodynamic trend among LIC, LSC, and LYC and not to quantitatively predict the products, reaction extent, or kinetics.

Notably, the evolution of the interfacial resistance and the formation of interphases governing this resistance can be systematically compared using the electrochemical four‐electrode cell. The viability of this method is demonstrated by the results of temperature‐dependent SXRD analysis and DFT calculations, opening a new avenue for further investigating the reaction kinetics and thermodynamics at the heteroelectrolyte interface and facilitating the construction of multilayer battery cells.

## 4 Conclusions

In this study, the electrochemical compatibility of three chloride SEs, LIC, LSC, and LYC, with the sulfide LPSI was investigated using electrochemical four‐electrode cells. The increase in the interfacial ion transport resistance at room temperature and the *E*_*a*_ values of the ion transport at the chloride | sulfide SE interfaces were successfully measured for the first time. The *E*_*a*_ value of Li_4_PS_4_I, which is strongly suggested to be the interfacial reaction product by high‐temperature SXRD measurements, corresponded to that for interfacial ion transport. This trend indicated that the interphase composed of the interfacial reaction products restricts interfacial ion transport and that Li_4_PS_4_I, which possesses high ionic conductivity, forms an ion‐transport pathway within the interphase.

The LIC|LPSI interface showed the highest interfacial resistance growth, followed by the LSC|LPSI and LYC|LPSI interfaces. The order of interfacial reactivity estimated by SXRD coincided with the order of the resistance increase rate, indicating that the difference in the growth rate of interfacial resistance was due to differences in the interfacial reaction rate. In addition, static 0 K DFT calculations reproduced the experimental order of the relative reactivity (LIC > LSC > LYC), although they do not quantitatively predict the reaction products or the reaction extent.

Notably, the interfacial reaction between the chloride and sulfide SEs at room temperature, which could not be detected by XRD measurements or by using electrochemical two‐electrode cells, could be detected using the electrochemical four‐electrode cell. This study demonstrates that interfacial resistance measurements using four‐electrode cells can aid the direct and sensitive evaluation of interfacial compatibility; therefore, the interfacial reaction products can be estimated by measuring the *E*_*a*_ value of the interfacial resistance.

## Funding

This study was supported by the Japan Society for the Promotion of Science (JP22H04608 and JP23K26762), the Japan Science and Technology Agency (JPMJAP2419 and JPMJSP2180), and the Toyota Physical and Chemical Research Institute.

## Conflicts of Interest

The authors declare no conflicts of interest.

## Supporting information

Supplementary Material

## Data Availability

The data that support the findings of this study are available from the corresponding author upon reasonable request.
