# Supplementary material for: Electrochemical Investigation of Compatibility Between Chloride‐ and Sulfide‐Based Solid Electrolytes Using Four‐Electrode Cells
Source: Small Sci. 2026 Oct 8;6(10):e70414. doi: 10.1002/smsc.70414 (PMC13650415; doi:10.1002/smsc.70414)
Supplement: Supplementary file 1 [file SMSC-6-e70414-s001.pdf]

Supporting Information for

"Electrochemical Investigation of Compatibility Between Chloride- and Sulfide-based Solid Electrolytes using Four-Electrode Cells"

Kotaro Yoshida[a], Atsunori Ikezawa[a,b,*], Peng Song[c], Shoma Yanagihara[d], Takeyoshi Okajima[a]
Saneyuki Ohno[c,d,e] and Hajime Arai[a]

[a] *School of Materials and Chemical Technology, Institute of Science Tokyo,*

Yokohama 226-8501, Japan

[b] *Department of Applied Chemistry for Environment, Tokyo Metropolitan University,*

1-1 Minami-osawa, Hachioji, Tokyo 192-0397 Japan

[c] *Institute of Multidisciplinary Research for Advanced Materials, Tohoku University,*

2-1-1 Katahira, Aoba-ku, Sendai, Miyagi 980-8577, Japan

[d] *Department of Applied Chemistry, Graduate School of Engineering, Kyushu University,*

744 Motooka, Nishi-ku, 819-0395 Fukuoka, Japan

[e] *Graduate School of Environmental Studies, Tohoku University,*

468-1 Aoba, Aoba-ku, Sendai, 980-8572, Japan

* Corresponding Author: (E-mail) ikezawa@mct.isct.ac.jp

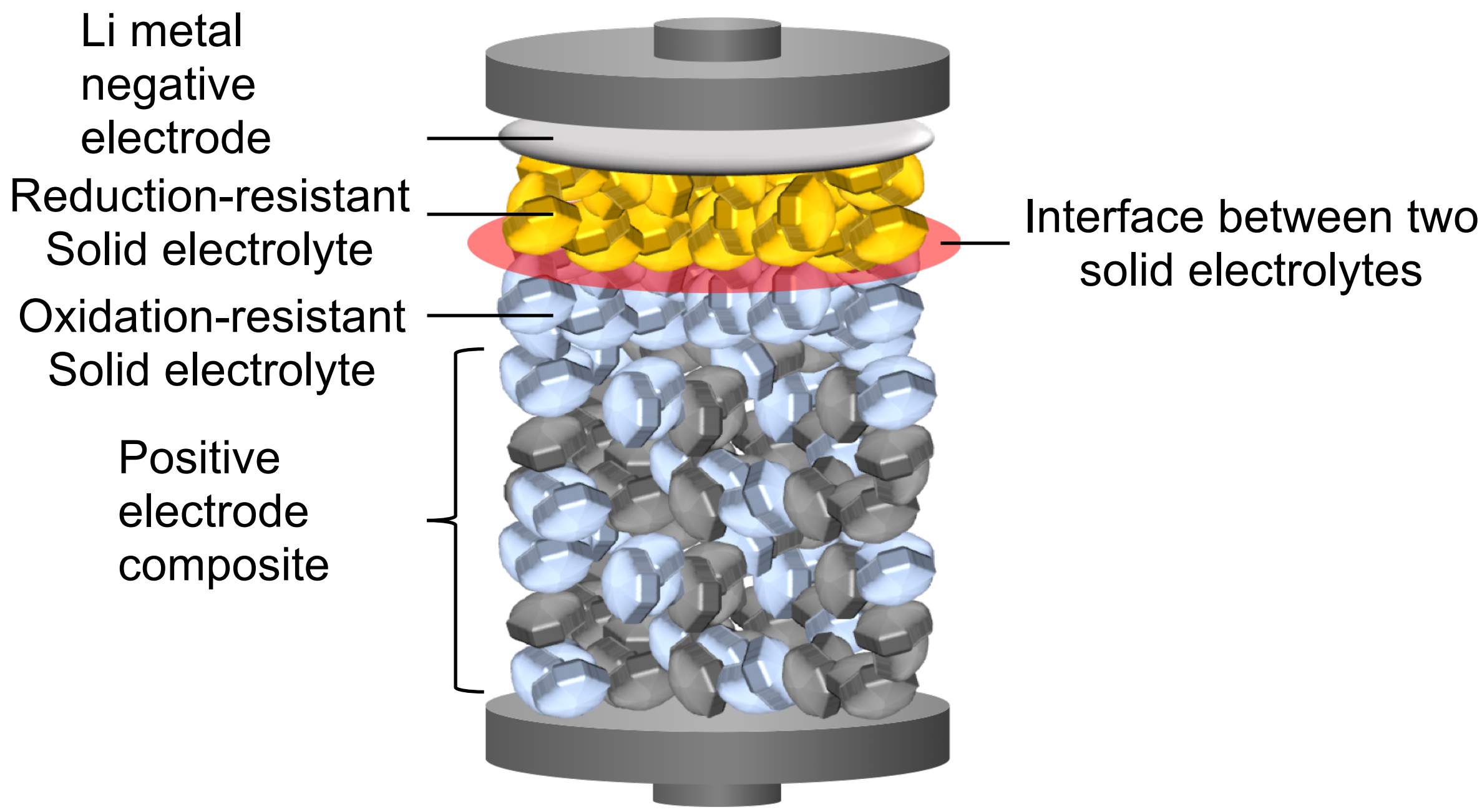

Figure S1. A schematic illustration of a hybrid bilayer cell. The oxidation-resistant solid electrolyte is mixed with the positive electrode materials, and the reduction-resistant solid electrolyte is placed in contact with the Li-metal negative electrode.

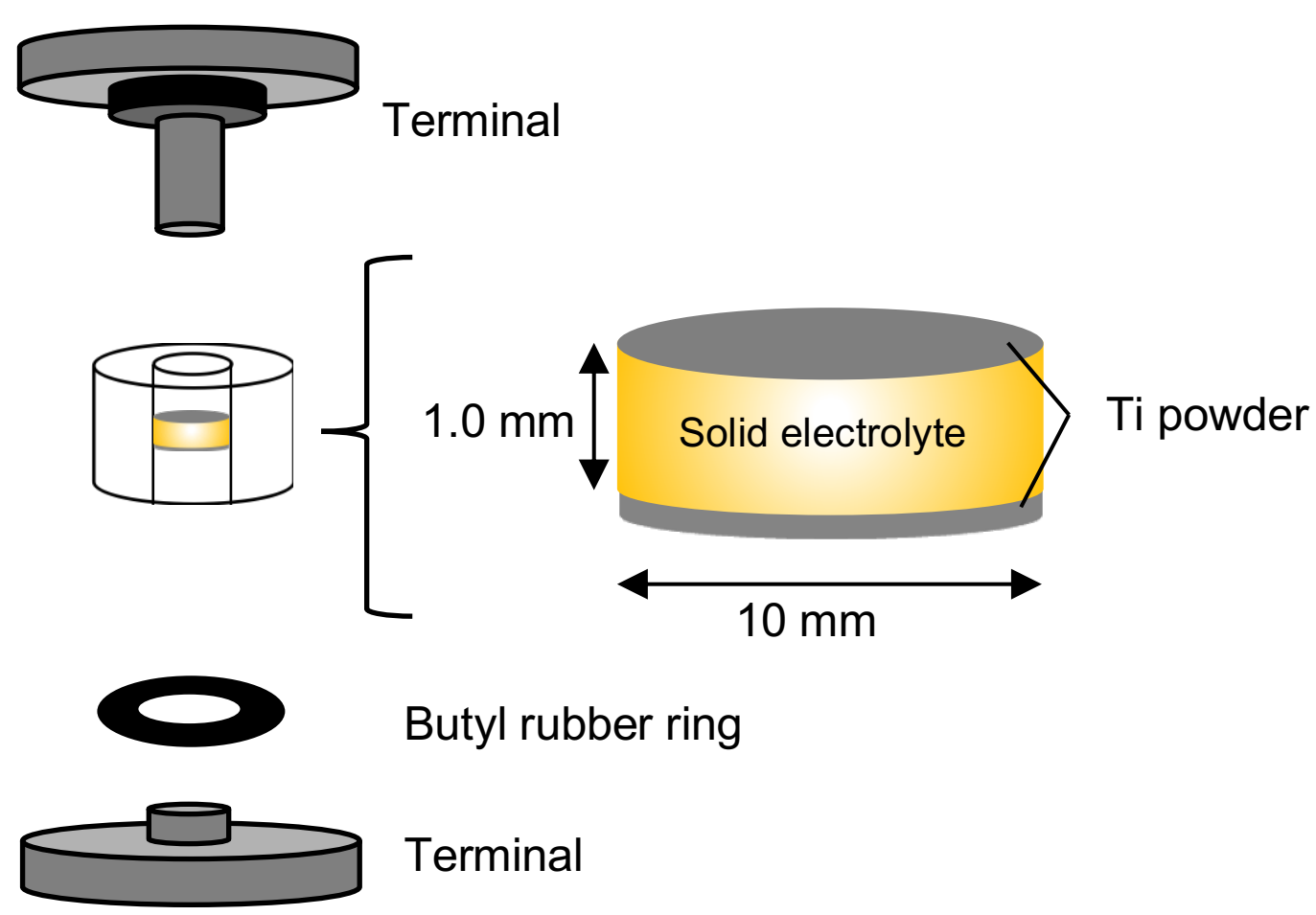

Figure S2. A schematic illustration of the two-electrode cells to measure the ionic conductivity of solid electrolytes. Titanium powder was pressed in contact with the solid electrolyte to form blocking electrodes.

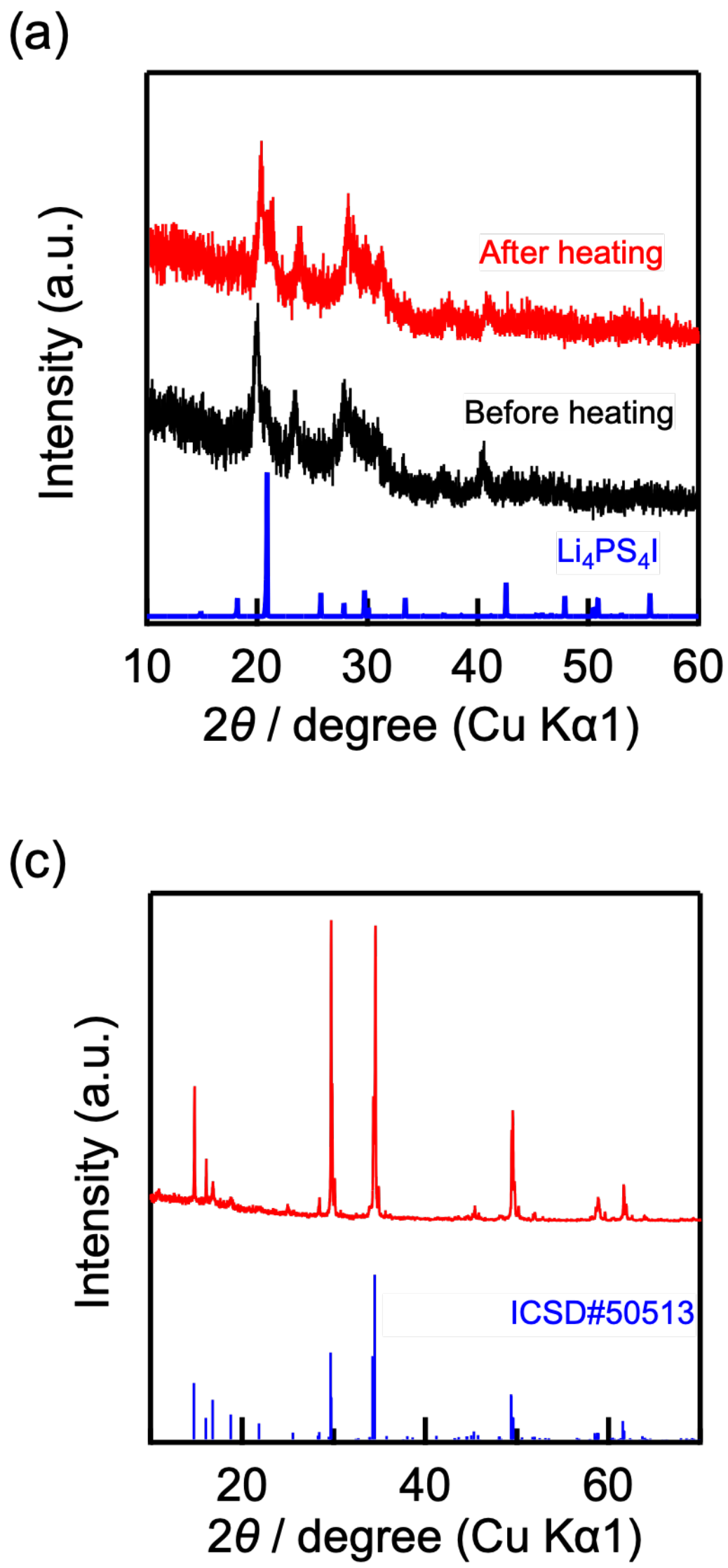

Figure S3. The XRD patterns of the (a) $Li_7P_2S_8I$ (before and after annealing at 448 K for 72 h), (b) Li_3InCl_6, (b) Li_3ScCl_6, (c) Li_3ScCl_6. The reference XRD pattern of Li_4PS_4I is simulated using reported crystal structure[1] and Z-Rietveld[2, 3].

Table S1. Measured and reported ionic conductivities (σ) and activation energies (E_a) of Li_3InCl_6, Li_3ScCl_6, Li_3YCl_6, and $Li_7P_2S_8I$.

	Li_3InCl_6	Li_3ScCl_6	Li_3YCl_6	$Li_7P_2S_8I$
σ at 298 K / mS cm^{-1}	0.36	0.5	0.28	2.7
E_a / eV	0.35	0.38	0.40	0.34
σ at 298 K / mS cm^{-1} (Reported values)	0.18–1.5[4, 5]	1.5[6]	0.12–0.51[5, 7]	1.35–1.57[8, 9]
E_a / eV (Reported values)	0.30–0.32[4, 5]	0.32–0.40[6]	0.34[5, 7]	0.40[8, 9]

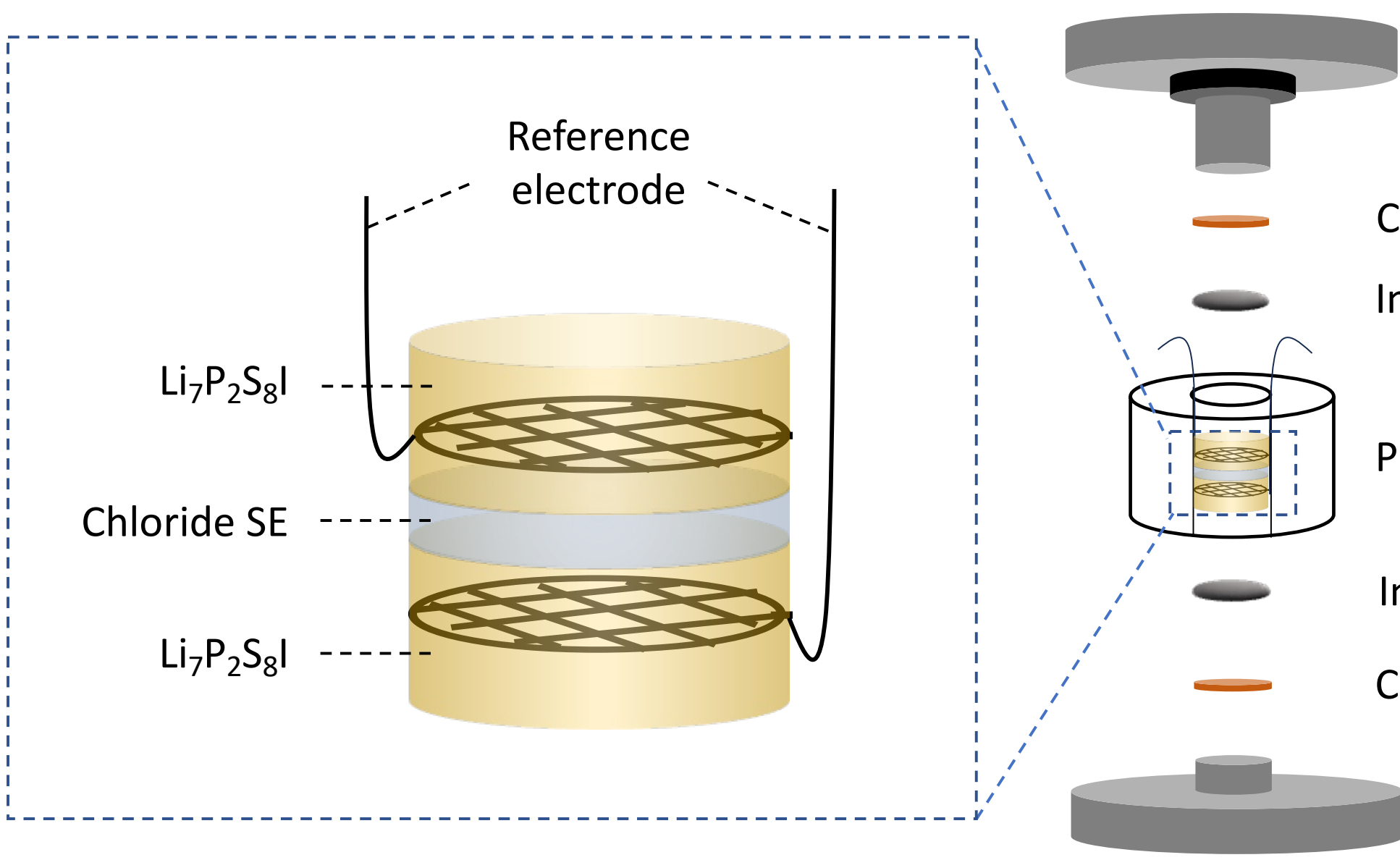

Figure S4. A schematic illustration of the configuration of an electrochemical four-electrode cell. A chloride solid electrolyte layer is sandwiched between $Li_7P_2S_8I$ to form an interface. The mesh-shaped R-LTO reference electrodes (RE) are embedded in $Li_7P_2S_8I$. Chemically-lithiated In-Li alloy foils are used as counter electrodes (CEs).

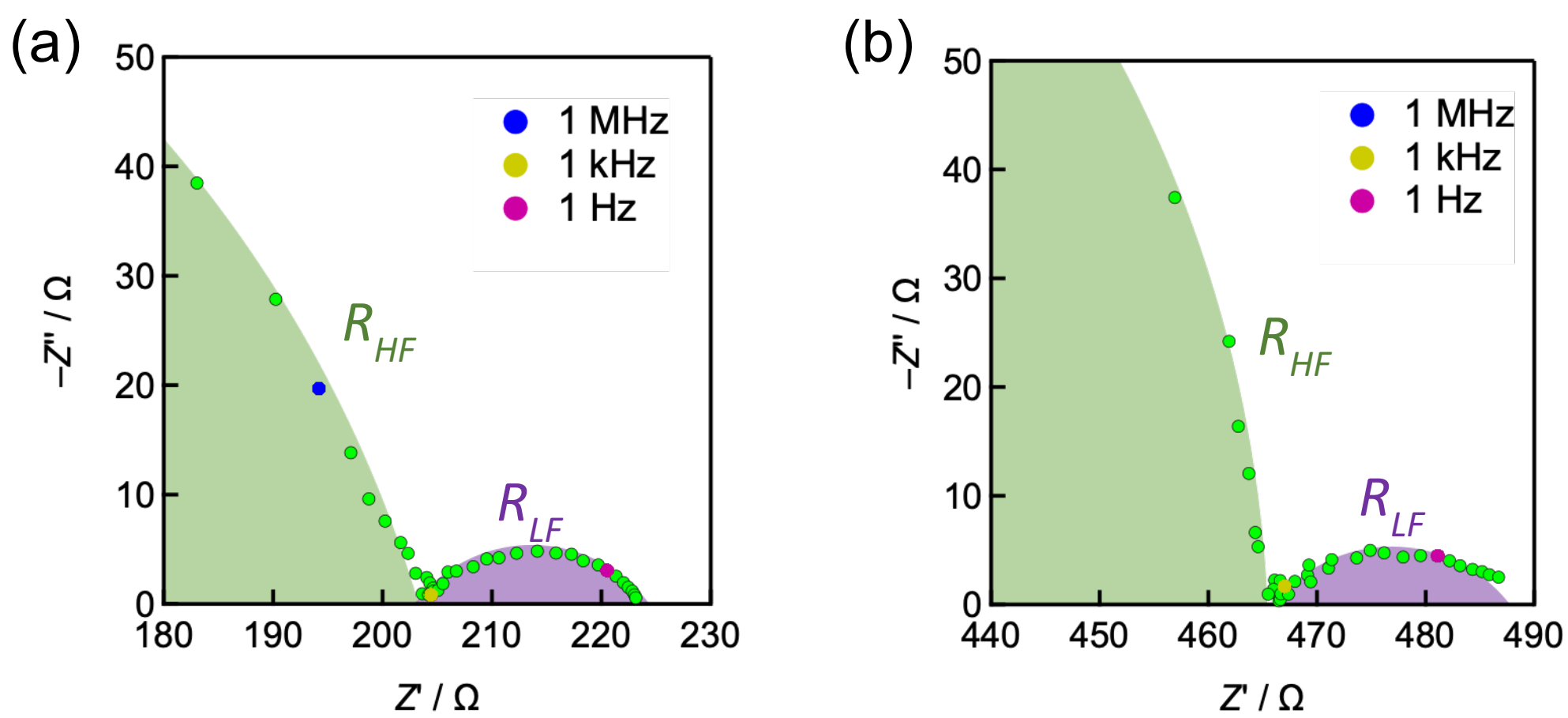

Figure S5. Impedance spectra of the (a) Li_3ScCl_6|$Li_7P_2S_8I$ cell and (b) Li_3YCl_6|$Li_7P_2S_8I$ cell.

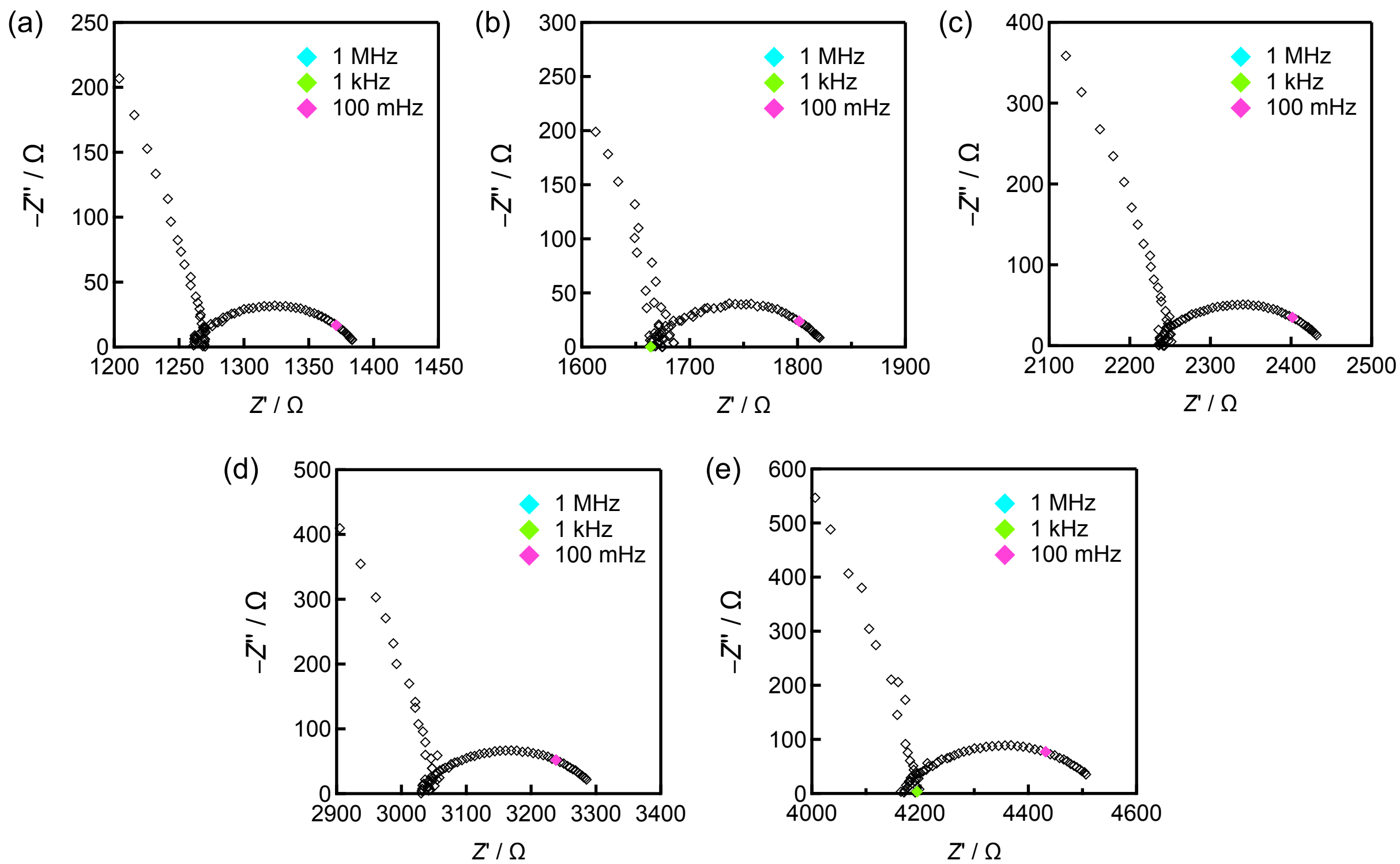

Figure S6. Impedance spectra of the Li_3InCl_6|$Li_7P_2S_8I$ cell at (a) 293 K, (b) 288 K, (c) 283 K, (d) 278 K, and (e) 273 K.

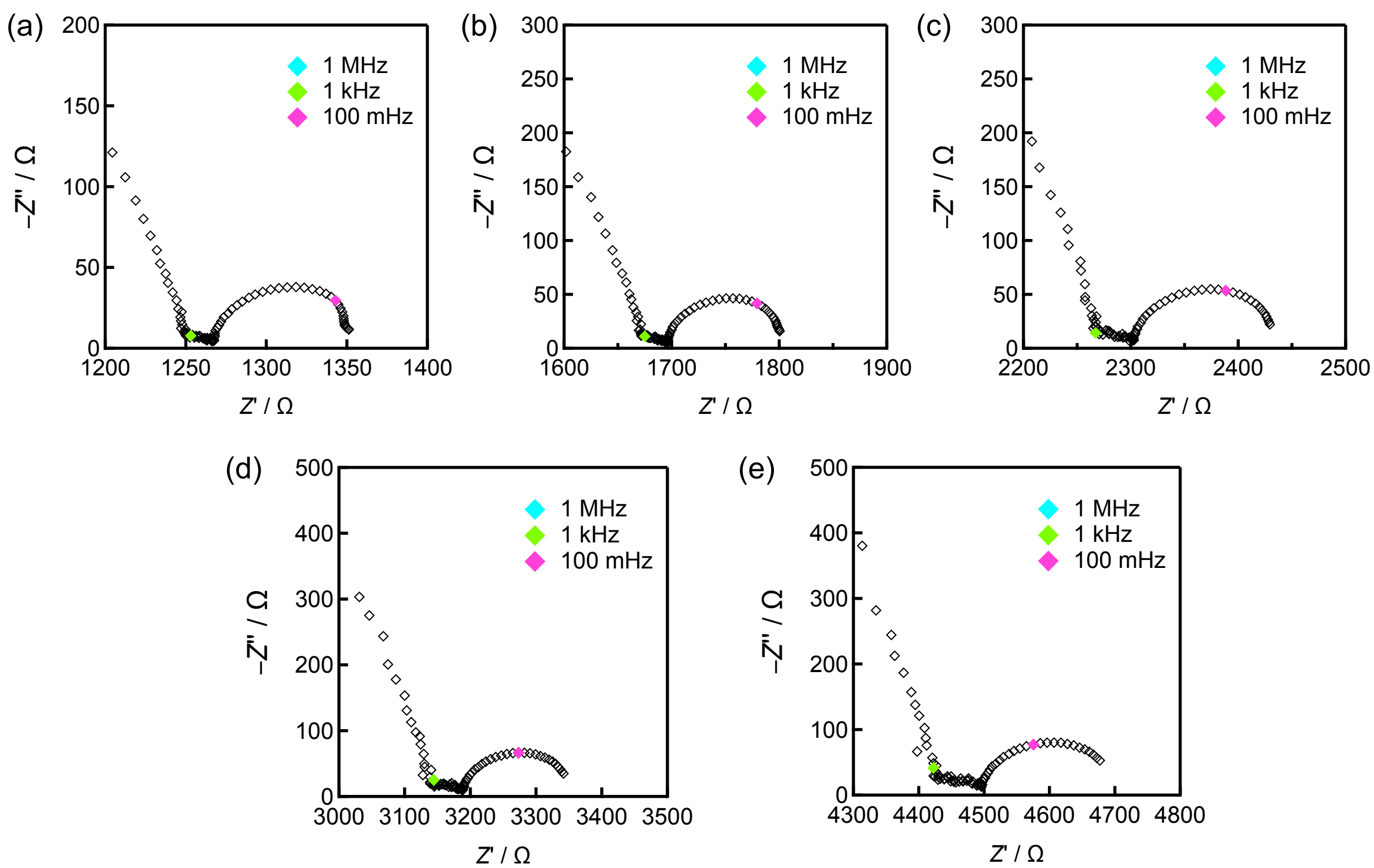

Figure S7. Impedance spectra of the $Li_3ScCl_6|Li_7P_2S_8I$ cell at (a) 293 K, (b) 288 K, (c) 283 K, (d) 278 K, and (e) 273 K.

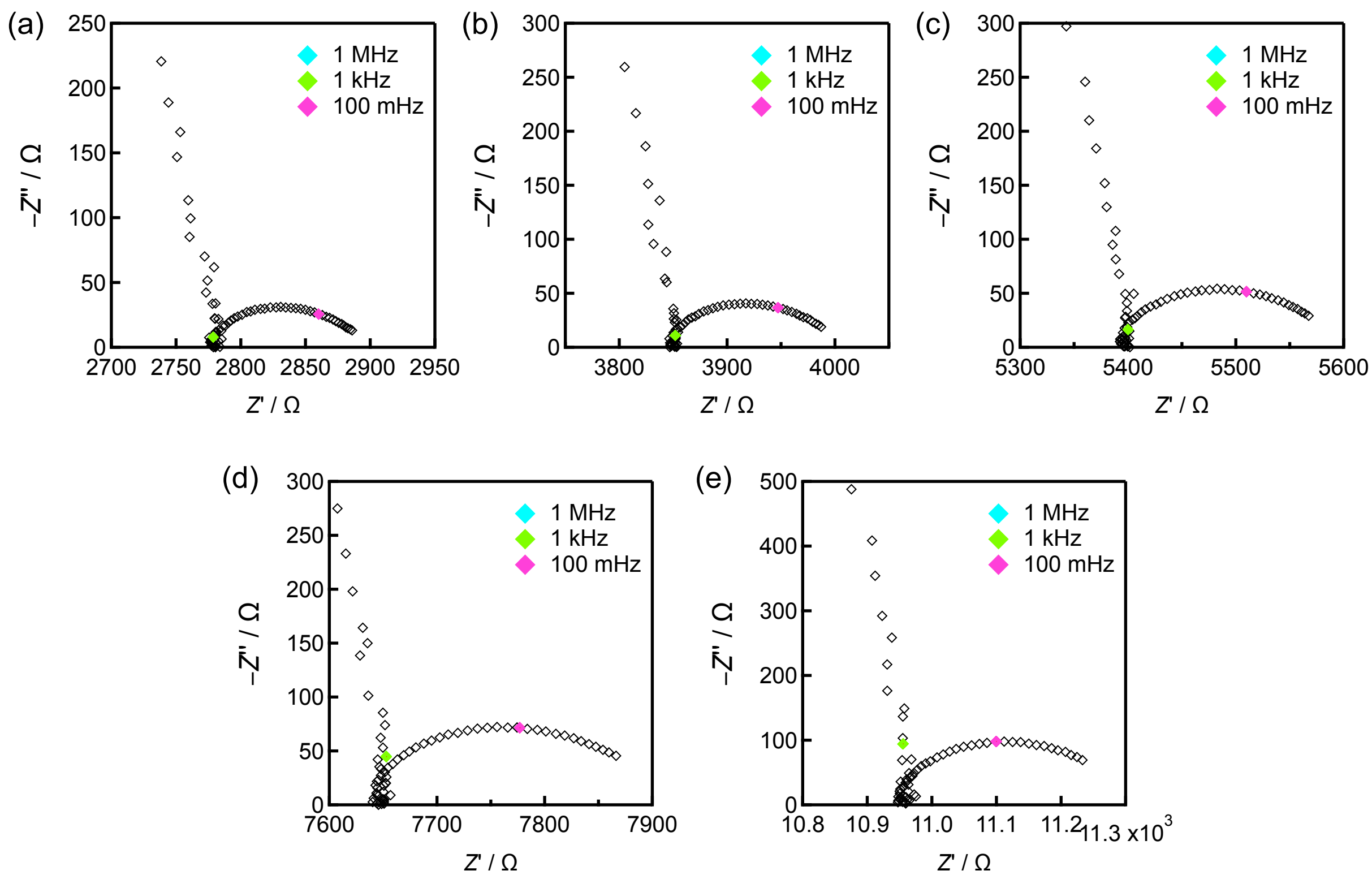

Figure S8. Impedance spectra of the $Li_3YCl_6|Li_7P_2S_8I$ cell at (a) 293 K, (b) 288 K, (c) 283 K, (d) 278 K, and (e) 273 K.

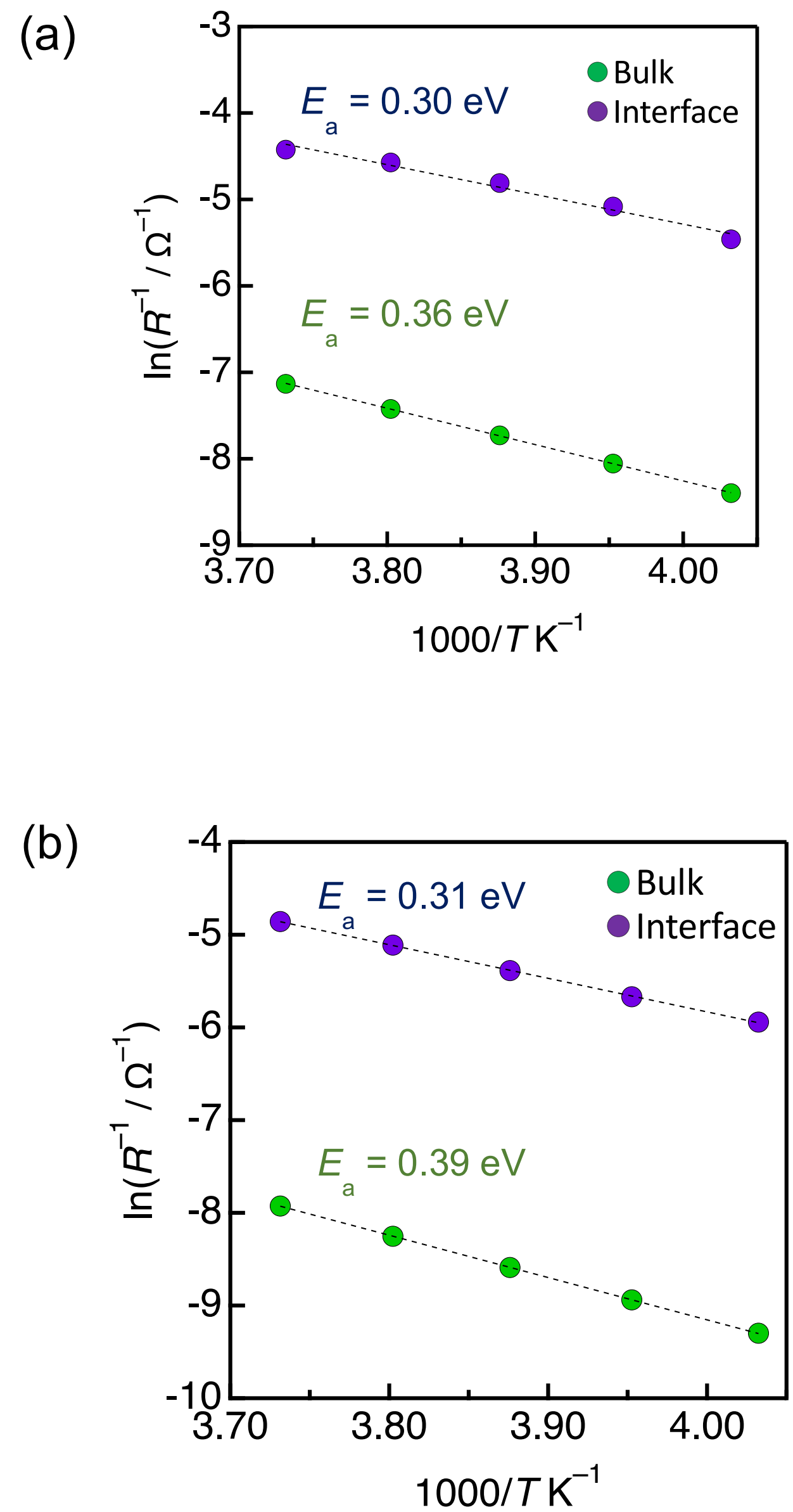

Figure S9. Arrhenius plots of R_{Bulk} and R_{Int} measured with (a) $Li_3ScCl_6|Li_7P_2S_8I$ cell and (b) $Li_3YCl_6|Li_7P_2S_8I$ cell.

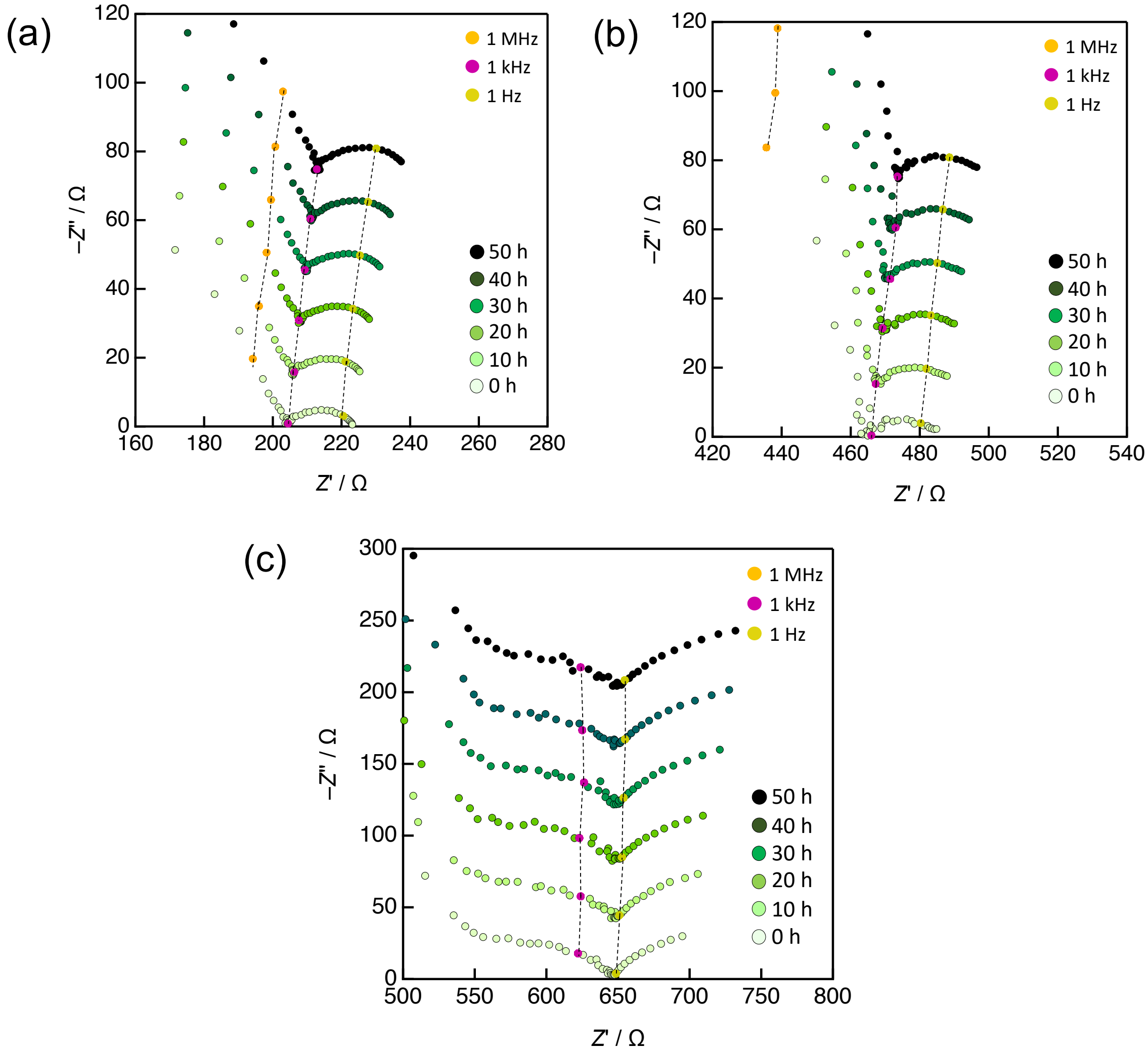

Figure S10. Time variation of the impedance spectra of the (a) $Li_3ScCl_6|Li_7P_2S_8I$, (b) $Li_3YCl_6|Li_7P_2S_8I$, and (c) $Li_7P_2S_8I$ $|Li_7P_2S_8I$ cells at room temperature (298 K).

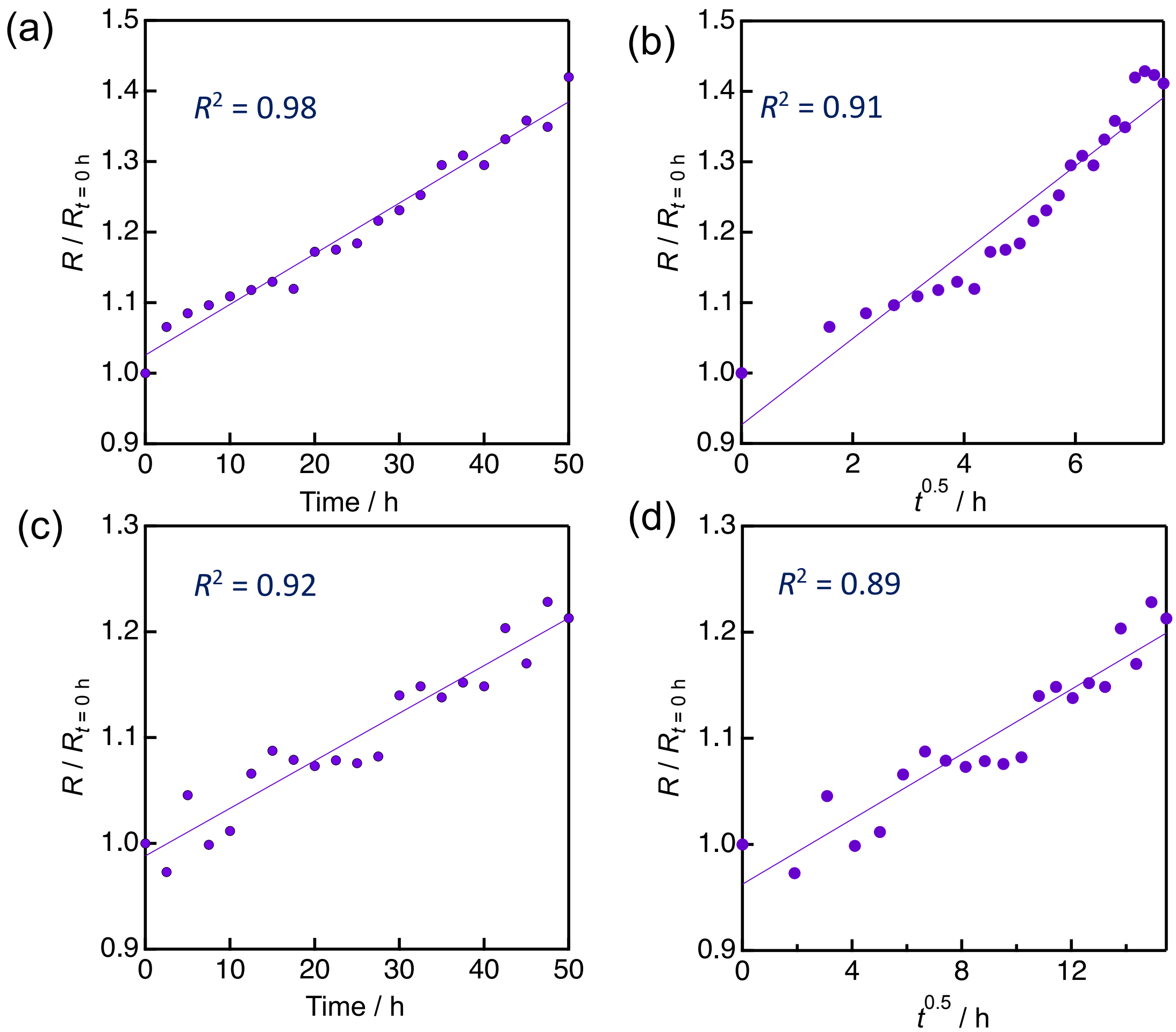

Figure S11. Interfacial resistance growth rates of the (a,b) LSC|LiPSI and (c,d) LYC|LPSI cells plotted against (a,c) time and (b,d) square root of time.

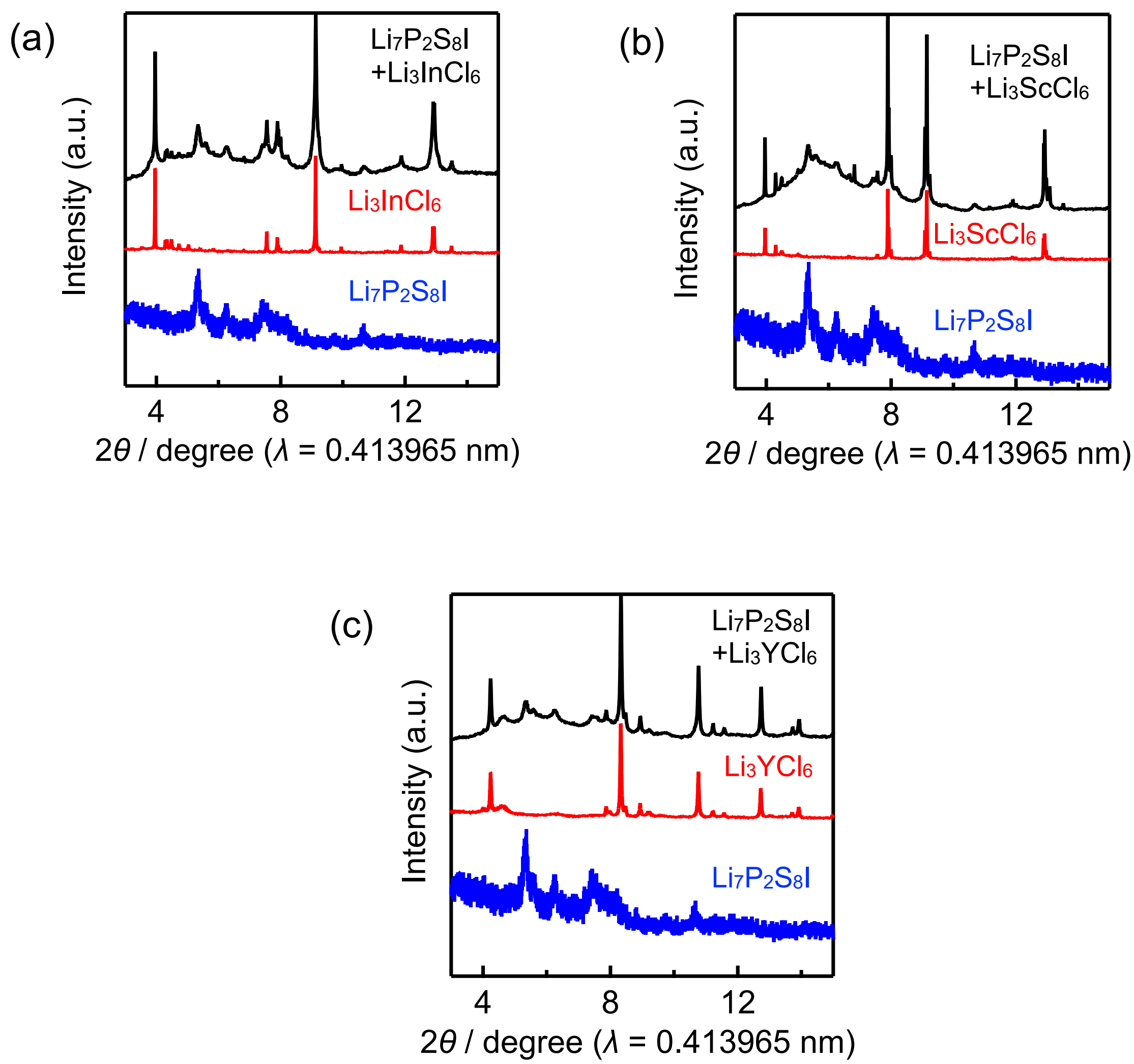

Figure S12. SXRD patterns of mixed powder of chloride SEs and $Li_7P_2S_8I$ left at 298 K for 1 weeks. XRD patterns of chloride SEs and LPSI using Cu Kα1 radiation converted to 2θ of SXRD are also presented. The SXRD patterns of the mixed powders only show the diffraction patterns of $Li_7P_2S_8I$ and chloride SEs.

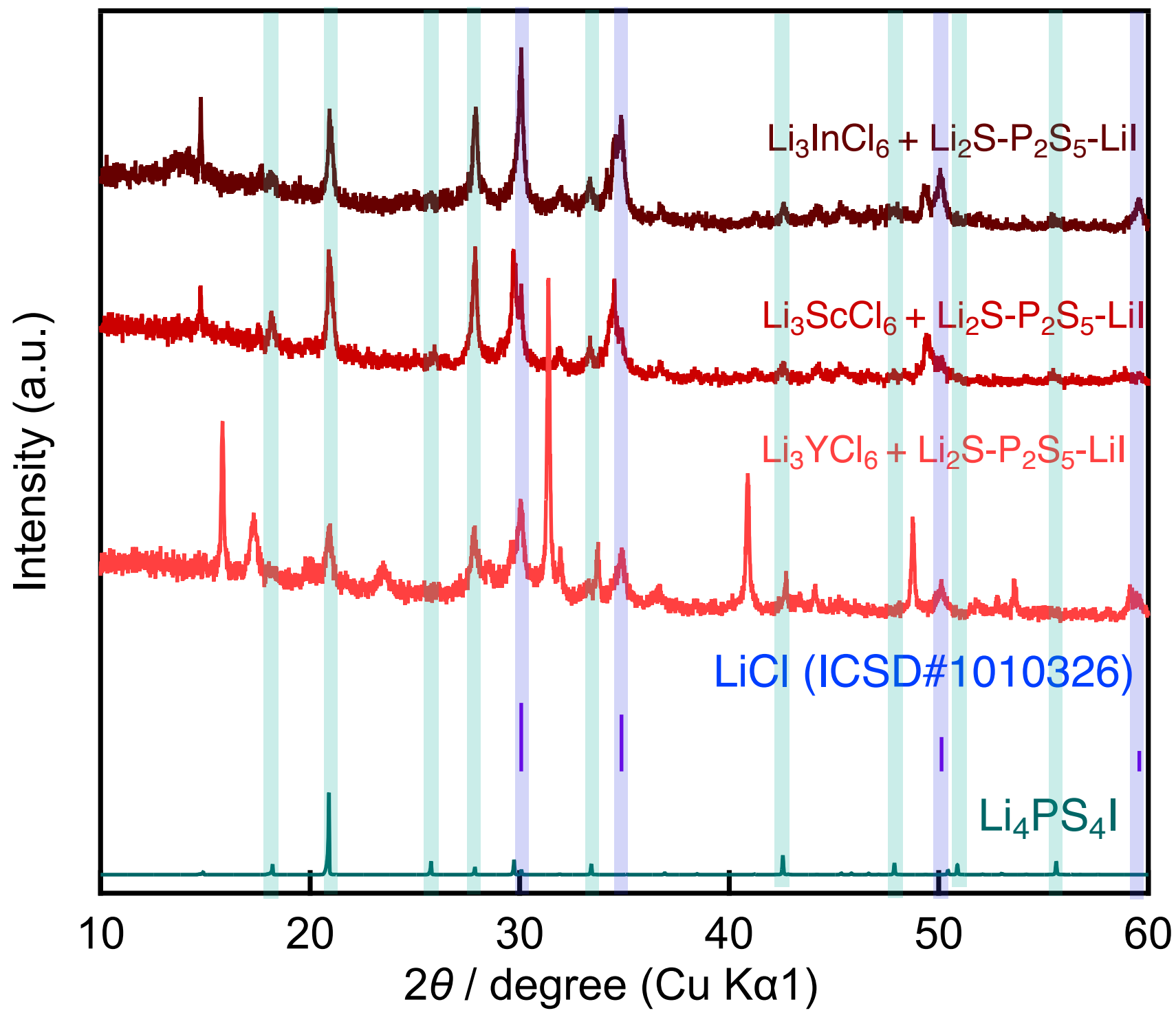

Figure S13. XRD patterns of Li_3InCl_6+$Li_7P_2S_8I$, Li_3ScCl_6+$Li_7P_2S_8I$, and Li_3YCl_6+$Li_7P_2S_8I$ after calcination at 423 K for 72 h.

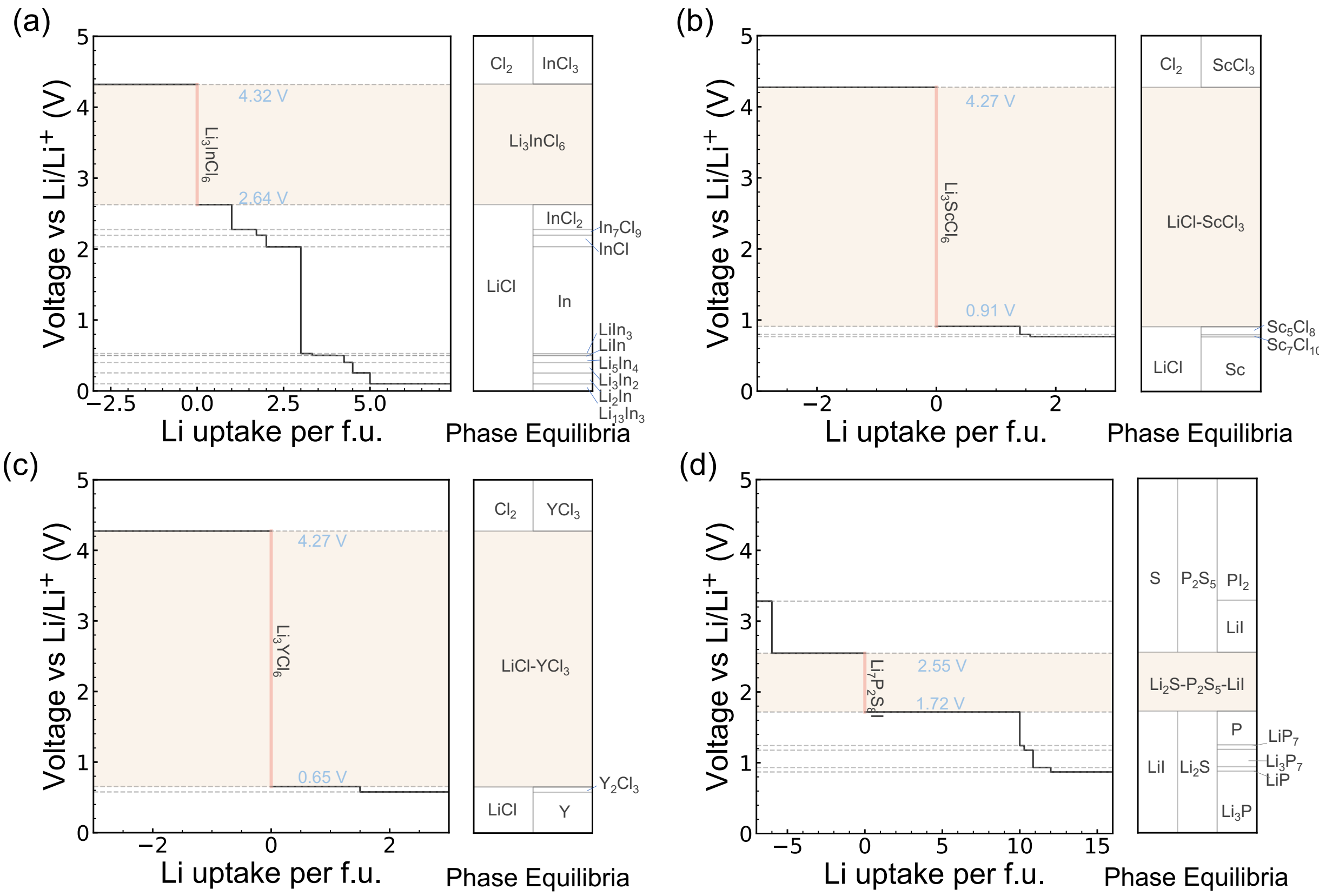

Figure S14. Phase equilibrium regions during lithiation (reduction) and delithiation (oxidation) for (a) LIC, (b) LSC, (c) LYC, and (d) LPSI.

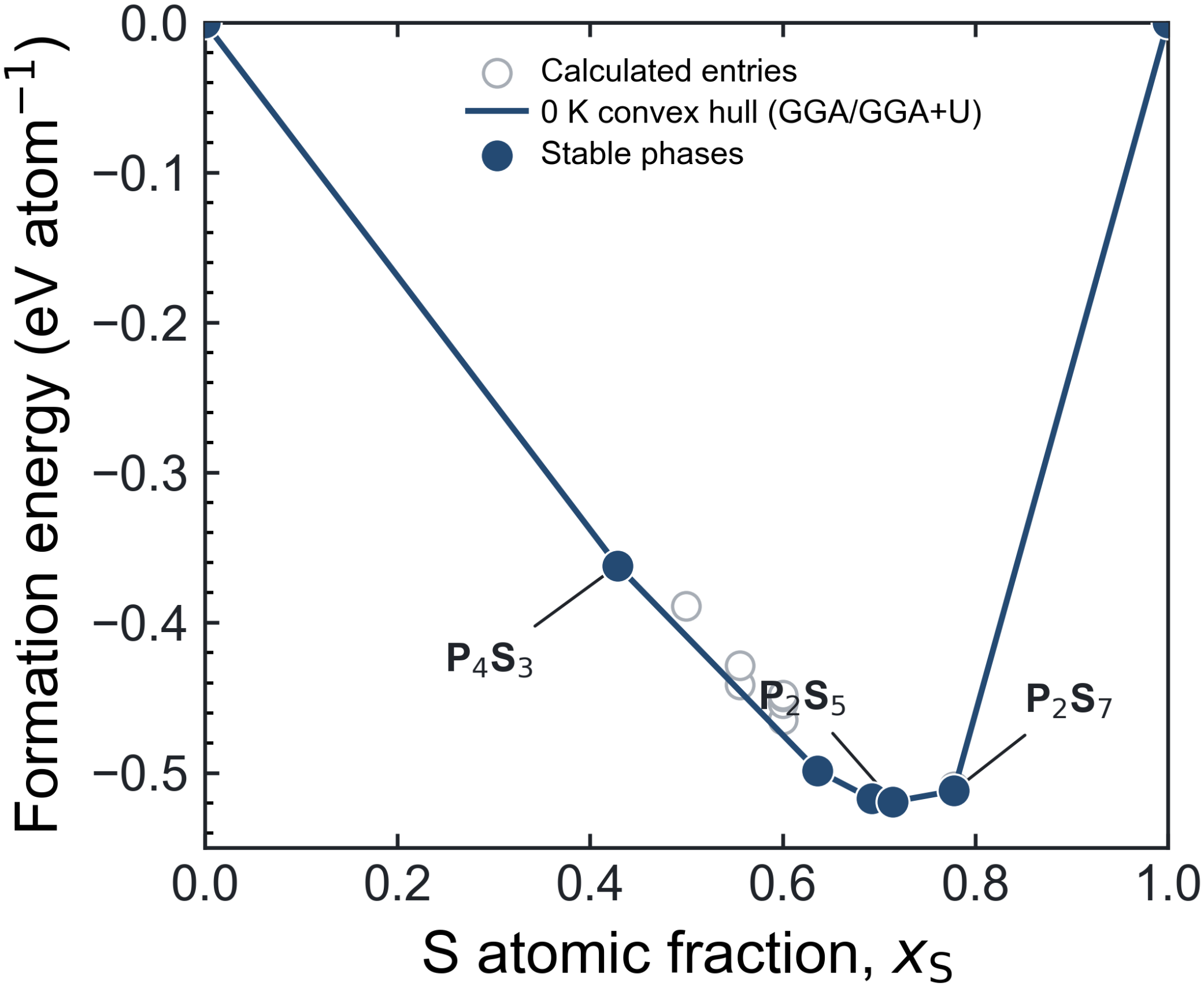

Figure S15. P–S convex-hull phase diagram at 0 K constructed using Materials Project GGA/GGA+U formation energies, consistent with the energy framework used for the interfacial-reaction analysis. P_4S_3, P_2S_5, and P_2S_7 are highlighted. The diagram provides phase-stability context only and does not represent a kinetic reaction pathway.

Table S2. Phase equilibria and minimum decomposition energies (ΔE_{chem}) at the interfaces between L*X*C and LPSI at the formula-unit-based molar fraction of L*X*C (x_m).

SSE1	SSE2	x_m	phase equilibria	ΔE_{chem} (eV/f.u.)
LPSI	LIC	0.50	$2\ Li_7P_2S_8I + 2\ Li_3InCl_6 = 12\ LiCl + P_2S_5 + In_2S_3 + 2\ Li_4PS_4I$	-0.68
		0.64	$4\ Li_7P_2S_8I$ + 7 Li3InCl6 = $42\ LiCl + 6\ LiInS_2 + 4\ P_2S_5 + LiInI_4$	-0.94
		0.65	$6\ Li_7P_2S_8I + 11\ Li_3InCl_6 = 66\ LiCl + 9\ LiInS_2 + 2\ InI_3 + 6\ P_2S_5$	-0.95
		0.71	5 Li_7P2S_8I + $12\ Li_3InCl_6 = 71\ LiCl + 2\ InI_2 + ICl + 10\ InPS_4$	-1.01
	LSC	0.43	$4\ Li_7P_2S_8I + 3\ Li_3ScCl_6 = 18\ LiCl + 3\ LiScS_2 + 2\ P_2S_5 + 4\ Li_4PS_4I$	-0.17
		0.50	$2\ Li_7P_2S_8I + 2\ Li_3ScCl_6 = 12\ LiCl + P_2S_5 + Sc_2S_3 + 2\ Li_4PS_4I$	-0.20
		0.60	$2\ Li_7P_2S_8I + 3\ Li_3ScCl_6 = 2\ LiI + 18\ LiCl + 3\ LiScS_2 + 2\ P_2S_5$	-0.24
		0.67	$Li_7P_2S_8I + 2\ Li_3ScCl_6 = LiI + 12\ LiCl + Sc_2S_3 + P_2S_5$	-0.27
	LYC	0.50	$2\ Li_7P_2S_8I + 2\ Li_3YCl_6 = 12\ LiCl + P_2S_5 + Y_2S_3 + 2\ Li_4PS_4I$	-0.07
		0.70	$3\ Li_7P_2S_8I + 7\ Li_3YCl_6 = 3\ Y_2S_3 + YI_3 + 42\ LiCl + 3\ P_2S_5$	-0.10

References

(1) Jodlbauer, A.; Spychala, J.; Hogrefe, K.; Gadermaier, B.; Wilkening, H. M. R. Fast Li Ion Dynamics in Defect-Rich Nanocrystalline Li_4PS_4I– The Effect of Disorder on Activation Energies and Attempt Frequencies. *Chem. Mater.* **2024**, *36* (3), 1648-1664. DOI: 10.1021/acs.chemmater.3c02988.

(2) Oishi, R.; Yonemura, M.; Nishimaki, Y.; Torii, S.; Hoshikawa, A.; Ishigaki, T.; Morishima, T.; Mori, K.; Kamiyama, T. Rietveld analysis software for J-PARC. *Nucl. Instrum. Methods Phys. Res. A* **2009**, *600* (1), 94-96. DOI: 10.1016/j.nima.2008.11.056

(3) Oishi-Tomiyasu, R.; Yonemura, M.; Morishima, T.; Hoshikawa, A.; Torii, S.; Ishigaki, T.; Kamiyama, T. Application of matrix decomposition algorithms for singular matrices to the Pawley method in Z-Rietveld. *J. Appl. Crystallogr.* **2012**, *45* (2), 299-308. DOI:10.1107/S0021889812003998

(4) Li, X. N.; Liang, J. W.; Luo, J.; Banis, M. N.; Wang, C. H.; Li, W. H.; Deng, S. X.; Yu, C.; Zhao, F. P.; Hu, Y. F.; et al. Air-stable Li3InCl6 electrolyte with high voltage compatibility for all-solid-state batteries. *Energ. Environ. Sci.* **2019**, *12* (9), 2665-2671. DOI: 10.1039/c9ee02311a.

(5) Yanagihara, S.; Huebner, J.; Huang, Z.; Inoishi, A.; Akamatsu, H.; Hayashi, K.; Ohno, S. Compatibility of Halide Electrolytes in Solid-State Li–S Battery Cathodes. *Chem. Mater.* **2025**, *37* (1), 109-118. DOI: 10.1021/acs.chemmater.4c02159.

(6) Zhou, L. D.; Kwok, C. Y.; Shyamsunder, A.; Zhang, Q.; Wu, X. H.; Nazar, L. F. A new halospinel superionic conductor for high-voltage all solid state lithium batteries. *Energ. Environ. Sci.* **2020**, *13* (7), 2056-2063. DOI: 10.1039/d0ee01017k.

(7) Asano, T.; Sakai, A.; Ouchi, S.; Sakaida, M.; Miyazaki, A.; Hasegawa, S. Solid Halide Electrolytes with High Lithium-Ion Conductivity for Application in 4 V Class Bulk-Type All-Solid-State Batteries. *Adv. Mater.* **2018**, *30* (44), 1803075. DOI: 10.1002/adma.201803075.

(8) Choi, S. J.; Lee, S. H.; Ha, Y. C.; Yu, J. H.; Doh, C. H.; Lee, Y.; Park, J. W.; Lee, S. M.; Shin, H. C. Synthesis and Electrochemical Characterization of a Glass-Ceramic Li7P2S8I Solid Electrolyte for All-Solid-State Li-Ion Batteries. *J. Electrochem. Soc.* **2018**, *165* (5), A957-A962. DOI: 10.1149/2.0981805jes.

(9) Wu, L. P.; Liu, G. Z.; Wan, H. L.; Weng, W.; Yao, X. Y. Superior lithium-stable Li7P2S8I solid electrolyte for all-solid-state lithium batteries. *J. Power. Sources.* **2021**, *491*, 229565. DOI: 10.1016/j.jpowsour.2021.229565.
